# Variation of body weight supported treadmill training parameters during a single session can modulate muscle activity patterns in post-stroke gait

**DOI:** 10.1007/s00221-023-06551-7

**Published:** 2023-01-13

**Authors:** Shraddha Srivastava, Bryant A. Seamon, Carolynn Patten, Steven A. Kautz

**Affiliations:** 1 Department of Health Sciences and Research, College of Health Professions, Medical University of South Carolina, 77 President Street, Charleston, SC 29425 USA; 2 Ralph H. Johnson VA Health Care System Medical Center, Charleston, SC 29401 USA; 3 Biomechanics, Rehabilitation, and Integrative Neuroscience (BRaIN) Lab, Department of Physical Medicine and Rehabilitation, University of California Davis School of Medicine, Sacramento, CA 95817 USA; 4 VA Northern California Health Care System, Martinez, CA 94553 USA; 5 Division of Physical Therapy, Department of Rehabilitation Sciences, Medical University of South Carolina, Charleston, SC 29425 USA

**Keywords:** Stroke, Locomotor training, Electromyography, Body weight support treadmill training, Muscle activation patterns, Individualized training

## Abstract

Evidence supporting the benefits of locomotor training (LT) to improve walking ability following stroke are inconclusive and could likely be improved with a better understanding of the effects of individual parameters i.e., body weight support (BWS), speed, and therapist assistance and their interactions with walking ability and specific impairments. We evaluated changes in muscle activity of thirty-seven individuals with chronic stroke (> 6 months), in response to a single session of LT at their self-selected or fastest-comfortable speed (FS) with three levels of BWS (0%, 15%, and 30%), and at FS with 30% BWS and seven different combinations of therapist assistance at the paretic foot, non-paretic foot, and trunk. Altered Muscle Activation Pattern (AMAP), a previously developed tool in our lab was used to evaluate the effects of LT parameter variation on eight lower-extremity muscle patterns in individuals with stroke. Repeated-measures mixed-model ANOVA was used to determine the effects of speed, BWS, and their interaction on AMAP scores. The Wilcoxon-signed rank test was used to determine the effects of therapist-assisted conditions on AMAP scores. Increased BWS mostly improved lower-extremity muscle activity patterns, but increased speed resulted in worse plantar flexor activity. Abnormal early plantar flexor activity during stance decreased with assistance at trunk and both feet, exaggerated plantar flexor activity during late swing decreased with assistance to the non-paretic foot or trunk, and diminished gluteus medius activity during stance increased with assistance to paretic foot and/or trunk. Therefore, different sets of training parameters have different immediate effects on activation patterns of each muscle and gait subphases.

**Supplementary Information:**

The online version contains supplementary material available at 10.1007/s00221-023-06551-7.

## Introduction

Stroke survivors frequently present with difficulty in walking. Thirty percent of community dwelling stroke survivors cannot walk independently (Lord et al. 2004). Stroke-impaired walking often reveals altered muscle activity patterns (Knutsson and Richards 1979, Shiavi et al. 1987, Den Otter et al. 2006, Srivastava et al. 2019), such as altered plantarflexor and gluteus medius activity during stance, which are associated with insufficient weight support (Allen et al. 2013) or altered co-excitation patterns of plantarflexors with other stance phase muscle activity associated with asymmetric walking (Brough et al. 2019). These altered patterns contribute to deviations in normal biomechanical functions of walking (Olney and Richards 1996; Srivastava et al. 2016) including: decreased gait speed (Nadeau et al. 1999), impaired balance control, gait asymmetry (Brough et al. 2019), and higher energy costs of walking (Olney and Richards 1996). Improving abnormal muscle activity patterns should thus be a major goal of rehabilitation to improve walking function.

Partial body weight support with treadmill walking has been found to modulate muscle activation patterns (Hesse et al. 1997; Routson et al. 2013). Body weight support (BWS) during walking results in immediate changes of individual muscle activity patterns (McGowan et al. 2008) and inter-muscular coordination (McGowan et al. 2010). Furthermore, greater change in BWS is associated with greater modulation (i.e. increase or decrease) of muscle activity in individuals with stroke (Hesse et al. 1997). Locomotor Training (LT) is a physical therapy intervention focused at improving walking function that includes stepping on the treadmill with partial BWS and therapist assistance (Duncan et al. 2011). LT has been shown to improve inter-muscular coordination associated with more symmetric gait and improved walking performance (Routson et al. 2013). However, the effects of LT on muscle activity patterns depend on the interaction of speed and BWS; for example, muscle activity is increased with greater walking speeds (Burnfield et al. 2016), but decreased with higher levels of BWS (Hesse et al. 1999). Together, these findings suggest LT could be used systematically to modify impaired muscle activity patterns (Routson et al. 2013; Burnfield et al. 2016) and potentially facilitate more normal walking patterns (Jørgensen et al. 1995) in individuals with stroke.

The various parameters of the LT paradigm (e.g., speed, level of BWS, type of assistance, etc.) influence different gait deficits (Chen et al. 2005; Chen and Patten 2006), moreover; given the myriad gait deficits revealed among stroke survivors, a uniform response to a certain set of LT parameters cannot be expected. In general, LT appears to be more effective in increasing walking speed of independent compared to non-independent walkers (Mehrholz et al. 2017). Increased BWS has been found to improve single limb support asymmetry (Chen et al. 2005) and faster walking speeds during LT to improve energy costs of walking (Hesse et al. 2001) in individuals with stroke. Furthermore, individuals with greater walking impairment improve with BWS at preferred walking speeds whereas individuals who have less impaired walking ability improve with BWS at fast walking speeds (Lamontagne and Fung 2004). There is evidence that very slow walking (i.e., < 0.4 m/s) involves a distinct motor pattern compared to walking at typical self-selected speed, suggesting that individuals training in this low speed range may be challenged to learn a new behavior rather than reestablishing an already learned, automatic behavior (Little et al. 2019). Additionally, the immediate response to therapist assistance at the paretic and non-paretic legs differs based upon the direction of baseline step length asymmetry (Little et al. 2020). Therefore, to develop an effective LT treatment program, it is important to understand the effects of individual training parameters and their interactions with the individual’s walking ability and their specific impairments.

While there has been extensive research on the effects of LT in the post-stroke population (Hesse et al. 1997, 1999; Chen and Patten 2006; Routson et al. 2013; Burnfield et al. 2016, Mehrholz et al. 2017), there is no consensus on its effects on gait rehabilitation. A large randomized controlled trial (LEAPS) demonstrating that improvements from LT were not superior to a home exercise program (Duncan et al. 2011) underscores a lack of clear evidence of efficacy of LT following stroke. This has led to the need for a more detailed understanding of the effects and interaction of LT parameters. Although, previous studies have looked at the effects of either the training speeds or BWS on impaired muscle activity (Hesse et al. 1999; Burnfield et al. 2016), there is no clear evidence on how speed, level of BWS, and type of assistance interact to modulate muscle activity patterns. We believe that the primary reason for this lack of information is the absence of a standardized metric to evaluate muscle activity during walking in stroke population. In the current study we systematically compare the effects of speed, level of BWS, and type of therapist assistance on muscle activity using the recently developed altered muscle activation patterns (AMAP) metric (Srivastava et al. 2019) during a single LT session in chronic stroke survivors. AMAP compares activity in individuals with stroke to that of similarly aged controls experiencing similar training conditions. We expect findings from the current study to help inform how various combinations of these parameters during a single session would produce muscle activity patterns in hemiparetic individuals that more closely resemble a healthy pattern. This information would direct future research on providing therapists with a rationale for selecting training parameters and on establishing the relationship between training parameters and increased benefits of LT.

## Methods

Forty individuals with chronic stroke (> 6 months) and 20 similarly aged controls with no neurological or orthopedic impairments, were studied during a single session of locomotor training (LT_SS_). Data from three stroke survivors were not included in the analysis due to incomplete data sets; of these two were unable to complete all the LT_SS_ parameters due to fatigue, and ground reaction force (GRF) data were missing for one stroke survivors. Individuals with stroke were included if they had a unilateral lesion; the ability to walk independently for at least 10 m at a self-selected speed ≤ 1.0 m/s; revealed no severe perceptual or cognitive deficits, no significant lower limb contractures, no severe osteoarthritis or prior pathological fracture, no significant cardiovascular impairments contraindicative to walking; were free of significant lower-extremity joint pain, range of motion limitations, and profound sensory deficits (including proprioception). Individuals who did not meet these criteria were excluded. Demographic characteristics are reported in Table 1. All participants provided informed consent approved by the University of Florida Health Science Center Institutional Review Board (IRB-01).

**Table 1:** Summary data for participant demographics

	Individuals with stroke	Controls
Sample size (*n*)	37	20
Age (years)	61.3 (11.5) [20–80]	(56 (9.2) [40–71])
Sex		
Female	10 (27%)	9 (45%)
Male	27 (73%)	11 (55%)
Lesion side		
Right	18 (48.7%)	N/A
Left	19 (51.3%)	
Months post-stroke	70.7 (62.5) [7–268]	N/A
Lower extremity Fugl-Meyer		
Total score	24 (4.9) [14–31]	N/A
Synergy score	16 (3.9) [8–22]	
Berg balance scale score	47 (4.7) [40–55]	N/A
Dynamic gait index score	14 (3.5) [7–22]	N/A
Overground walking speed (m/s)		
Self-selected	0.64 (0.19) [0.29–1.14]	(1.33 (0.2) [0.97–1.72])
Fastest comfortable	0.94 (0.36) [0.29–1.92]	(1.91 (0.23) [1.39–2.3])

Continuous variables are presented as mean (standard deviation) [range]. Categorical variables are presented as a frequency count (percentage)

### Data acquisition

Controls were studied while walking on a treadmill under a BWS system (Therastride, St. Louis MO) in nine conditions presented in randomized order. Manipulation variables included three speeds (0.3, 0.6, and 0.9 m/s) and three levels of BWS (0%, 15%, and 30%). Individuals with stroke walked on the treadmill at their self-selected and fastest-comfortable walking speed at three levels of BWS (0%, 15%, and 30%). Since stroke survivors with a self-selected speed ≤ 1.0 m/s were included in the study, we used pre-defined speeds i.e., at 0.3, 0.6, 0.9 m/s for our healthy participants to match their walking speeds with those of stroke survivors. A safety harness attached to the Constant Force Body Weight Support system mounted to the laboratory ceiling was worn by participants to provide BWS for the experimental conditions and also protect them in the event of loss of balance. Thus, they were not allowed to use the handrails for additional support. Individuals with stroke were allowed to adjust their self-selected and fast walking speeds at 15% and 30% BWS if it changed due to the increased BWS. Additionally, all stroke survivors completed 7 conditions at their fastest speed with 30% BWS and therapist assistance presented in randomized order. Conditions with therapist assistance consisted of paretic foot (P), non-paretic foot (N), trunk (T), paretic foot with trunk (PT), non-paretic foot with trunk (NT), both feet (PN), and both feet with trunk (PNT). One 30-s trial per condition was conducted for stroke survivors and controls. All participants were allowed as much rest as needed between trials, but not less than 2 min. Therapist assistance for the paretic and non-paretic foot was designed to promote step length symmetry with therapists’ hands placed at the ankle. Therapists assisted with trunk movement in the mediolateral direction to promote weight-shift symmetry. All LT_SS_ parameters were delivered by licensed physical therapists, who had previously gained experience with the standardized protocol while working on several other research studies conducted at the VA Brain Rehabilitation Research Center and the University of Florida Health Science Center.

Electromyographic (EMG) data were collected from 8 muscles in both legs (MA-416-003 Motion Lab System Baton Rouge, LA) including: tibialis anterior (TA), soleus (SO), medial gastrocnemius (MG), vastus medialis (VM), rectus femoris (RF), lateral hamstring (LH), medial hamstring (MH), and gluteus medius (GM). Here we present EMG data for the paretic leg for stroke survivors and bilaterally for controls. GRF were captured using two force plates embedded in the instrumented split-belt treadmill (Bertec Corp., Columbus, OH, USA). EMG and ground reaction force data were sampled at 2000 Hz except for one stroke survivor where data were accidentally sampled at 1000 Hz.

### Altered muscle activation patterns metric

The AMAP metric was used to evaluate deviations in EMG amplitude and timing patterns between individuals with stroke and controls during walking (Srivastava et al. 2019). The AMAP accounts for spatiotemporal gait asymmetries commonly present in stroke and typical variability within the healthy population. EMG data were high pass filtered (20 Hz) with a zero lag fourth-order Butterworth filter, demeaned, rectified, and smoothed with a zero lag fourth-order low-pass (25 Hz) Butterworth filter. The EMG data from each muscle were then normalized to the averaged peak activation across all the steps taken by the subject at their self-selected walking speed. *K*-means cluster analysis was used to identify the “on” and “off” periods of EMG activity (Den Otter et al. 2007).

To evaluate the EMG activity during walking, we divided the gait cycle into six regions: first double support (DS1), first (SS1) and second halves of single-leg stance (SS2), second double support (DS2), and first (SW1) and second halves of swing (SW2). Within each of these regions, AMAP scores were computed for timing and amplitude components of the EMG activity. The timing component was equal to the “on” time within each region expressed as a percentage of the total duration of that region:

1 $${\text{Timing}}\;{\text{Component}}_{{{\text{HEALTHY}}}} = \left( {\frac{{{\text{On}}\;{\text{time}}\;{\text{for}}\;{\text{region}} }}{{{\text{Total}}\;{\text{time}}\;{\text{for}}\;{\text{region}}}}} \right)*100$$

The amplitude component was equal to the integrated EMG i.e., net area under the curve (AUC) of the “on” time for each region expressed as a percentage of the total integrated EMG of all “on” periods of the gait cycle:

2 $${\text{Amplitude}}\;{\text{Component}}_{{{\text{HEALTHY}}}} = \left( {\frac{{{\text{Integrated}}\;{\text{EMG}}\;{\text{amplitude}}\;{\text{during}}\;{\text{On}}\;{\text{time}}\;{\text{of}}\;{\text{a}}\;{\text{region }}}}{{{\text{Total}}\;{\text{Integrated}}\;{\text{EMG}}\;{\text{amplitude}}\;{\text{of}}\;{\text{the}}\;{\text{gait}}\;{\text{cycle}}}}} \right)*100 $$

Equations (1) and (2) were applied to all healthy individuals and means were computed for the timing and amplitude components in each of the six regions for the eight muscles for each trial condition. AMAP is based on the *z*-scores. A *z*-score is a measure of the number of standard deviations an element falls above or below the population mean. In the current study means and standard deviations of healthy controls within each speed and BWS condition for both the amplitude and timing components of each region were used as normal patterns (Eqs. 3, 4, respectively) to compute the AMAP scores for individuals with stroke at the corresponding speed and BWS of each LT_SS_ condition.

3 $${\text{Amplitude}}\;{\text{Component}}\;{\text{AMAP}}\;{\text{Score}}_{{{\text{STROKE}}}} = \frac{{{\text{Amplitude}}\;{\text{Component}}_{{{\text{STROKE}}}} - {\text{ Amplitude}}\;{\text{Component}}\;{\text{Mean}}_{{{\text{HEALTHY}}}} }}{{{\text{Amplitude}}\;{\text{Component}}\;{\text{SD}}_{{{\text{HEALTHY}}}} }}$$

4 $${\text{Timing Component AMAP Score}}_{{{\text{STROKE}}}} = \frac{{{\text{ Timing Component}}_{{{\text{STROKE}}}} - {\text{ Timing Component Mean}}_{{{\text{HEALTHY}}}} { }}}{{{\text{Timing Component SD}}_{{{\text{HEALTHY}}}} }}$$

Amplitude and Timing component AMAP Score_STROKE_ represent the scores of each stroke survivor for a given LT_SS_ condition; Amplitude and Timing component Mean_HEALTHY_ represents the group mean of healthy individuals walking at speeds and BWS similar to individuals with stroke; Amplitude and Timing component SD_HEALTHY_ represents group SD of healthy individuals walking at speeds and BWS similar to individuals with stroke. Calculation of AMAP scores for stroke survivors requires comparison to mean and SD of healthy individuals at similar speed and BWS (Mean_HEALTHY_ and SD_HEALTHY_). Thus, based on the training speed of a stroke survivor (i.e., self-selected or fast walking speeds during 15% and 30% BWS) we used means (Mean_HEALTHY_) and standard deviations (SD_HEALTHY_) of controls walking without therapist assistance at speeds of 0.3 m/s (for training speeds < 0.4 m/s—i.e., household ambulators), 0.6 m/s (for training speeds between 0.4 and 0.8 m/s—i.e., limited community ambulators) and 0.9 m/s (for training speeds > 0.8 m/s—i.e., community ambulators) at corresponding BWS (15% or 30%) to compute AMAP scores in Eqs. (3) and (4). During training, each stroke survivor was allowed to adjust their self-selected and fastest walking speeds with increased BWS. If walking speed changed for a stroke survivor when BWS increased, they were reassigned to the new group and EMG patterns were compared with healthy controls at walking speeds and BWS matched to the new group values. Matching speed and weight support of stroke survivors with healthy controls at each training parameter improves the ability of our analytical tool to evaluate the deviation in EMG activity of hemiparetic gait relative to normal, because biomechanical demands change with walking speed and weight support (Kirtley et al. 1985; McGowan et al. 2008; Little et al. 2019) requiring a change in the associated muscle activity. We defined *z*-scores ranging between ± 2.57 as “normal” (Srivastava et al. 2019). For statistical analysis, we used absolute values of the *z*-scores for amplitude and timing components. AMAP scores were averaged across gait cycles within each of the six regions (i.e., DS1, SS1, SS2, DS2, SW1, and SW2). Therefore, our analysis produced an amplitude and a timing score of each stroke survivor for each of the 8 muscles and walking conditions for each of the six regions.

The AMAP scores for timing and amplitude quantify EMG patterns of stroke survivors relative to those of similarly aged control participants and their range of typically observed variability. The AMAP scores for each condition was computed by comparing EMG patterns of individuals with stroke to that of healthy individuals during similar walking conditions. An AMAP score closer to zero would suggest that EMG activity is closer to that of healthy individuals’ walking at similar speed and BWS and vice versa. Note that an increase in the EMG activity of individuals with stroke following LT_SS_ could result in either a worse score, if it shifts further from zero and becomes less similar to normal outside the bounds of normal variability for that muscle within that region, or an improved score, if it shifts closer to zero and becomes more similar to normal. Therefore, the effect of increases and decreases in EMG activity depends on how that change compares to the change in activity observed in the similar healthy condition. AMAP scores of stroke survivors (i.e., EMG patterns of stroke survivors computed relative to the EMG pattern of healthy controls during similar walking conditions) for each LT_SS_ condition were compared with their baseline AMAP scores to evaluate the response to each training parameter.

#### Statistics

A repeated-measures mixed-model ANOVA was used to determine the effects of speed, BWS, and their interaction, on AMAP scores during walking with 0%, 15% or 30% BWS at self-selected or fast walking speeds without therapist assistance. To meet normality assumptions required for mixed-model comparisons, if the AMAP scores were not normally distributed they were first log transformed and if normality assumptions were still not met, were square root transformed across all conditions to improve model fit for each muscle’s amplitude and/or timing component. Statistical significance was set a priori at *p* ≤ 0.05, with Bonferroni-correction used to correct for multiple comparisons. Mean and SD values of untransformed AMAP scores are reported in Table 1 of supplementary material. The Wilcoxon-signed rank test was used to compare AMAP scores between each of therapist-assisted conditions and baseline self-selected walking at 0% BWS. For each of the Wilcoxon-signed rank tests, statistical significance adjusted for multiple comparisons was set at *p* < 0.007. Mean and SD values of AMAP scores for each condition are reported in Table 2 of supplementary material. All statistics were performed in SPSS version 25 (IBM Co., Somers, NY) and SAS version 9.4 (SAS Institute Inc., Cary, NC).

## Results

*Baseline Patterns (AMAP scores of individuals with stroke at self-selected walking speed with no BWS or therapist assistance):* We derived AMAP scores for EMG amplitude and timing of individuals with stroke at self-selected walking speed relative to controls’ EMG patterns at similar speeds. At this baseline, AMAP scores of individuals with stroke revealed exaggerated activity during DS1 and SW2 and diminished activity during SS2 for SO and MG. We also observed exaggerated activity for TA during DS2 and for GM during DS2, SW2 and diminished activity for GM during SS1. In most stroke survivors, AMAP patterns for MH, LH, RF, and VM were not dissimilar from controls, meaning they fell within the range of typically observed variability. The data used for baseline scores have been reported in our previous work defining the AMAP (Srivastava et al. 2019). These baseline AMAP scores were computed with EMG patterns of individuals with stroke at self-selected walking speed without BWS or therapist assistance and healthy controls’ EMG pattern at 0.3 m/s, 0.6 m/s, and 0.9 m/s walking speeds without BWS or therapist assistance.

*Speed effect:* The predominant effect of speed was to cause plantarflexor activation earlier in the gait cycle resulting in increased total activity during stance. There were significant main effects of speed for SO amplitude scores during SS1 [*F* (1, 124) = 5.35, *p* = 0.0224], DS2 [*F* (1, 124) = 7.24, *p* = 0.0081], and for SO timing scores during DS1 [*F* (1, 124) = 4.39, *p* = 0.0383], SW2 [*F* (1, 124) = 4.45, *p* = 0.0370]. Significant main effects of speed were also observed for MG amplitude scores [*F* (1, 106) = 6.20, *p* = 0.0143] and timing scores [*F* (1, 106) = 5.48, *p* = 0.0211] during DS1 and for amplitude scores only during SS2 [*F* (1, 106) = 12.93, *p* = 0.0005]. Specifically, relative to baseline AMAP scores, increased speed revealed worse SO amplitude scores (“worse” means more abnormal AMAP scores) during SS1 (i.e., increased amplitude) and DS2 (i.e., decreased amplitude). Increased speed also led to worse SO timing scores during DS1 and SW2 (i.e., longer “on” time). Similarly, increased speed led to worse MG amplitude scores during DS1 and SS2 (i.e., increased amplitude), and worse timing scores during DS1 (i.e., longer “on” time). At baseline, both SO and MG amplitude are diminished in individuals with stroke during stance phase, but here an increase in amplitude with speed results in worse AMAP scores because it exceeded the level of normal activity seen in healthy controls. There were no significant effects of speed on MH activity patterns. For the remainder of the significant changes observed in each muscle pattern, the AMAP scores were within the normal range at baseline and remained within the normal range (i.e., normal variability of healthy controls) following LT_SS_ conditions involving speed change.

*BWS effect:* Increasing the amount of BWS (which decreased the biomechanical demands for individual muscles to provide support) mostly tended to improve muscle activation. Significant main effects of weight were seen for SO AMAP scores during DS1 [amplitude: *F* (2, 124) = 10.40, *p* < 0.0001; timing: *F* (2, 124) = 10.74, *p* < 0.0001], SS1 [amplitude: *F* (2, 124) = 12.62, *p* < 0.0001; timing: *F* (2, 124) = 5.01, *p* = 0.0080], and SW2 [amplitude: *F* (2, 124) = 11.27, *p* < 0.0001; timing: *F* (2, 124) = 20.99, *p* < 0.0001]; for MG AMAP scores during SW2 [amplitude: *F* (2, 106) = 6.57, *p* = 0.0020; timing: *F* (2, 106) = 5.41, *p* = 0.0058], and DS1 [amplitude: *F* (2, 106) = 4.66, *p* = 0.0115]. Significant main effects of weight were also seen for TA AMAP scores during DS2 [timing: *F* (2, 123) = 18.72, *p* < 0.0001], for GM AMAP scores during SS1 [amplitude: *F* (2, 122) = 26.71, *p* < 0.0001; timing: *F* (2, 122) = 8.37, *p* = 0.0003], DS2 [amplitude: *F* (2, 122) = 14.48, *p* < 0.0001; timing: *F* (2, 122) = 7.74, *p* = 0.0007], SS2 [amplitude: *F* (2, 122) = 6.03, *p* = 0.0032] and SW1 [amplitude: *F* (2, 122) = 5.07, *p* = 0.0076]. Specifically, the AMAP scores for SO improved with increased BWS relative to baseline scores during DS1 and SW2 (i.e., decreased amplitude and shorter “on” time) and during SS1 (i.e., increased amplitude and longer “on” time). With increased BWS, AMAP scores for MG amplitude during DS1 were worse (i.e., increased amplitude). MG amplitude and timing scores improved during SW2 (i.e., decreased amplitude and shorter “on” time). AMAP scores improved significantly with increased BWS for the TA timing component during DS2 (i.e., longer “on time”). The scores improved during SS1 and SS2 for GM amplitude and during SS1 for timing (i.e., increased amplitude and longer “on” time). There was also an improvement in scores for GM amplitude during DS2 and SW1 (i.e., decreased amplitude), and for timing during DS2 (i.e., shorter “on” time). For the remainder of the significant changes observed in each muscle pattern the AMAP scores were within the normal range at baseline and remained within the normal range (i.e., normal variability of healthy controls following LT_SS_ conditions involving BWS changes).

*Interaction of speed and BWS:* There were no significant interaction effects.

**Fig. 1:**
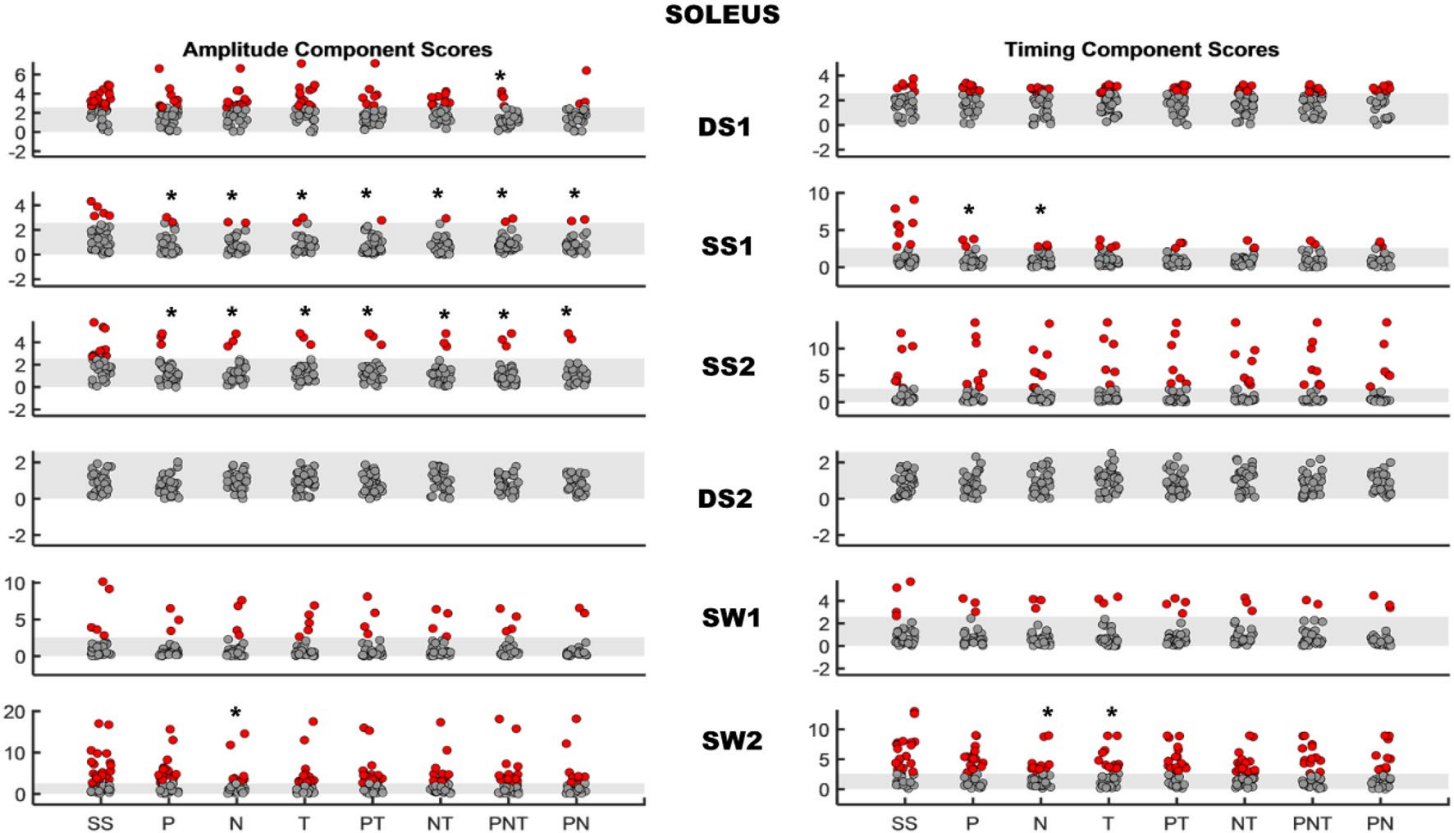
AMAP scores of Soleus in stroke survivors. Left panel: amplitude component for each therapist-assisted condition, right panel: timing component for each therapist-assisted condition. Each row represents a region of gait cycle. The shaded gray area is the normal range of scores (± 2.57). Each dot within a region of gait cycle represents the score of a stroke survivor with solid red dots representing participants with scores outside the normal window of ± 2.57. Asterisks indicate conditions where AMAP scores were significantly different from the baseline scores

**Fig. 2:**
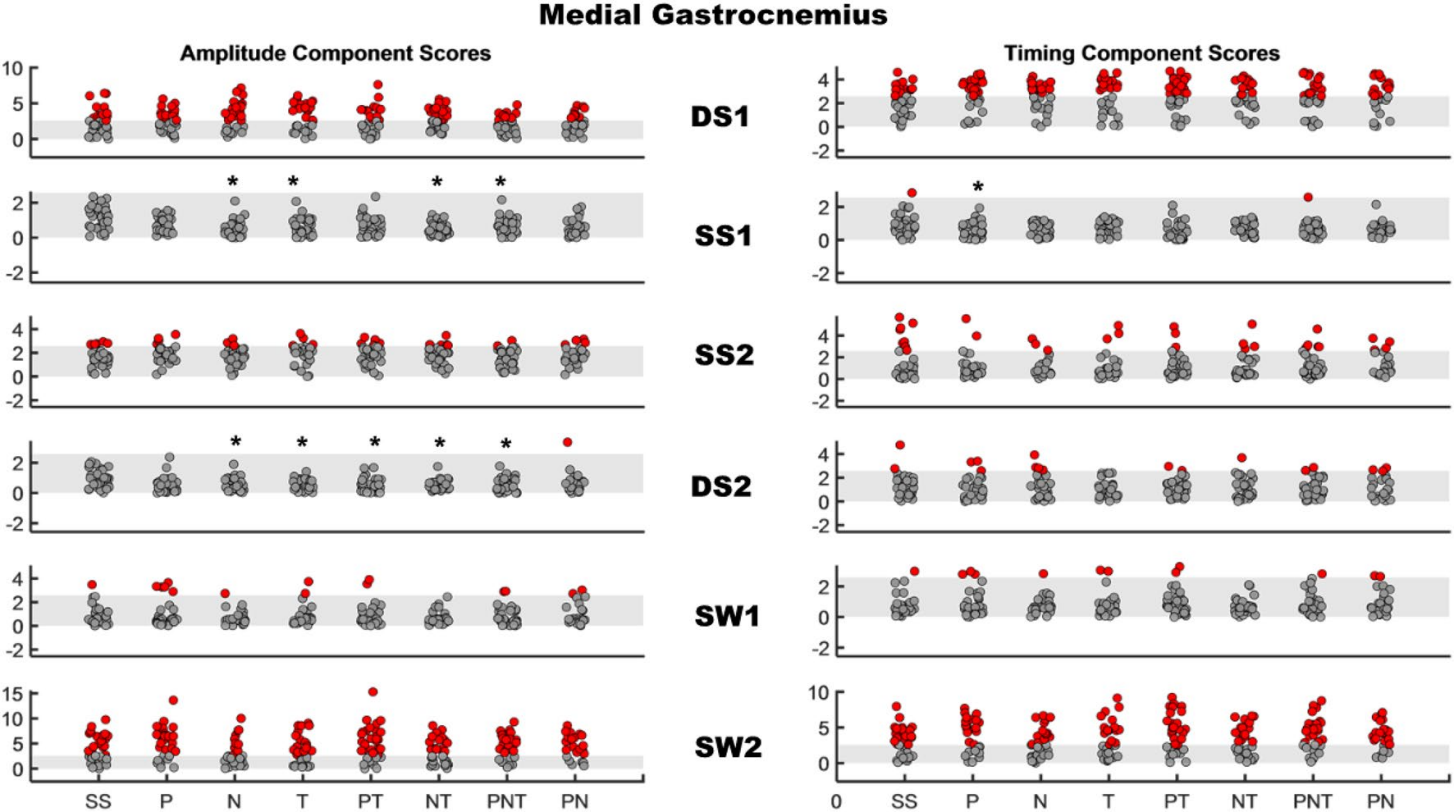
AMAP scores of Medial Gastrocnemius in stroke survivors. Left panel: amplitude component for each therapist-assisted condition, right panel: timing component for each therapist-assisted condition. Each row represents a region of the gait cycle. The shaded gray area is the normal range of scores (± 2.57). Each dot within a region of the gait cycle represents the score of a stroke survivor with solid red dots representing participants with scores outside the normal window of ± 2.57. Asterisks indicate the condition where AMAP scores were significantly different from the baseline scores

**Fig. 3:**
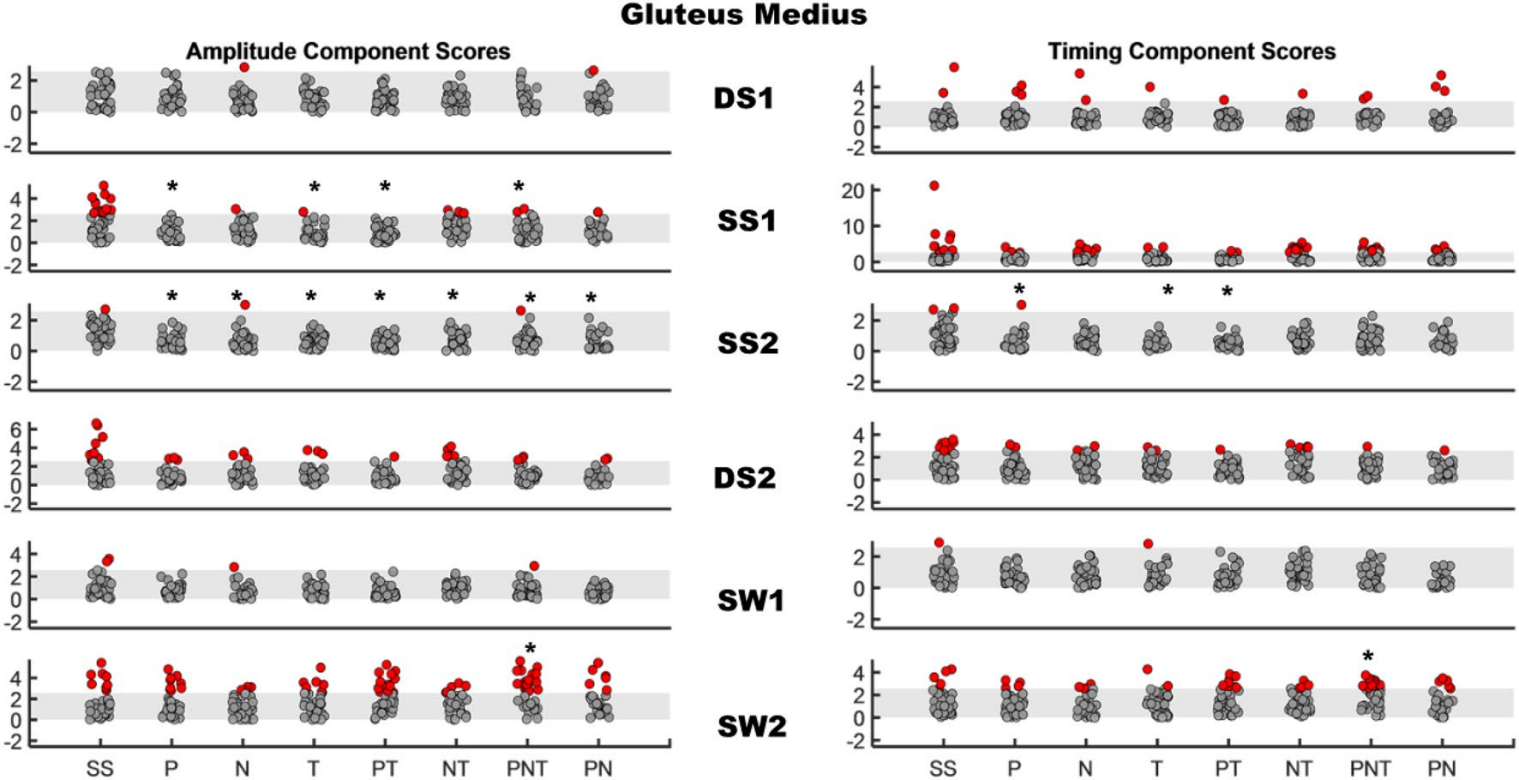
AMAP scores for Gluteus Medius in stroke survivors. Left panel: amplitude component for each therapist-assisted condition, right panel: timing component for each therapist-assisted condition. Each row represents a region of gait cycle. The shaded gray area is the normal range of scores (± 2.57). Each dot within a region of the gait cycle represents the score of a stroke survivor with solid red dots representing participants with scores outside the normal window of ± 2.57. Asterisks indicate the condition where AMAP scores were significantly different from the baseline scores

*Effects of therapist assistance at 30% BWS and fast walking speeds:* Significant changes with therapist assistance were seen mostly for timing and amplitude scores in SO, MG, and GM activity. SO amplitude AMAP scores improved significantly relative to baseline (i.e., increased amplitude) during SS1 and SS2 for all LT_SS_ parameters and during DS1 following trunk and both feet assistance. SO amplitude AMAP scores improved significantly during SW2 (i.e., decreased amplitude) following non-paretic foot assistance (Fig. 1). The SO timing AMAP scores improved only with paretic or non-paretic foot assistance during SS1 (i.e., longer “on” time) and with non-paretic foot assistance or trunk assistance during SW2 (i.e., shorter “on” time) (Fig. 1). For MG, AMAP amplitude scores improved during SS1 (i.e., increased amplitude) following assistance to non-paretic foot, trunk, trunk and non-paretic foot, or trunk and both feet (Fig. 2). The AMAP scores for MG timing during DS1 became worse (i.e., longer “on” time) with assistance to the paretic foot (Fig. 2). The AMAP amplitude scores for GM became worse during SW2 (i.e., increased amplitude) with therapist assistance to trunk and both feet, and AMAP timing scores became worse during SW2 (i.e., longer “on” time) with assistance to trunk and both feet (Fig. 3). The AMAP scores for GM amplitude improved (i.e., increased amplitude) during SS1 with therapist assistance for paretic foot, trunk, trunk and paretic foot, or trunk and both feet and during SS2 with all therapist-assisted conditions (Fig. 3). The GM timing component demonstrated significant improvement of AMAP scores during SS2 (i.e., longer “on” time) with therapist assistance to paretic foot, trunk, or trunk and paretic foot. The AMAP scores for TA amplitude activity became worse (i.e., increased amplitude) during SS1 with assistance to trunk and both feet and during DS2 with assistance to non-paretic foot or trunk. No significant changes were seen in the AMAP timing component for TA. Baseline activity as well as significant changes with training parameters fell within the normal range for MH, LH, RF, and VM. *P*-values are reported in Table 3 of supplementary material.

## Discussion

The current study evaluated immediate effects of locomotor training parameters on muscle activation patterns during a single session by varying the levels of body weight support, walking speed, and several combinations of therapist assistance at the trunk, paretic foot, and non-paretic foot. We compared muscle activation patterns of stroke survivors with similarly aged controls evaluated at similar speeds and BWS (i.e., 0.3 m/s, 0.6 m/s or 0.9 m/s, at BWS 0%, 15% or 30%) to identify the combination of LT_SS_ parameters that resulted in muscle activation patterns most similar to normal walking. Most of the parameter variation effects were observed for the SO, MG, and GM activity patterns (Figs. 1, 2, 3), which are the primary muscles that showed abnormal baseline activity following stroke (Srivastava et al. 2019). We found that increasing BWS to 30% improved the EMG patterns of SO, MG, and GM. We also found that therapist assistance to the trunk and both feet with 30% BWS improved plantarflexor activity during stance and assistance to the non-paretic foot and trunk improved plantarflexor activity during swing. Therapist assistance to the trunk and paretic foot improved GM patterns during stance but worsened them during swing. Furthermore, contrary to the previous literature, we found that increases in speed resulted in worse plantarflexor activity. Collectively our findings revealed that different parameters of LT_SS_ target different deficits of muscle activity patterns, and not all LT_SS_ parameters may be beneficial towards improving muscle activity in individuals with stroke. This information has the potential to direct future research to inform clinicians on training parameters to effect desired changes in activation patterns of different muscles and gait sub-phases focused at individually tailored gait rehabilitation.

### Faster speeds may not be favorable to improve plantarflexor patterns

Increased speed resulted in worse amplitude and timing AMAP scores seen as increased activity (i.e., greater than healthy controls) for SO and MG. A previous study evaluating the effects of BWS training at different speeds demonstrated that plantarflexor activity at faster speeds was greater than at slower speeds (Burnfield et al. 2016). However, increased EMG activity with speed would not necessarily mean an activity pattern closer to normal. More recently van Kammen et al. demonstrated that all individuals with stroke walking with BWS and robotic assistance had higher peak EMG amplitude, closer to healthy individuals, walking on treadmill at 0.56 m/s compared to 0.28 m/s (van Kammen et al. 2020); but, with increased speeds they did not find any improvements in temporal step symmetry which is associated with plantarflexor function (Allen et al. 2011). EMG activity was evaluated within each gait subphase in comparison to healthy individuals, but unlike the current study where AMAP evaluated both the amplitude and timing of EMG activity patterns, van Kammen et al., evaluated only the peak activity. It is indeed possible that faster walking increases peak EMG amplitude, without normalizing the overall muscle activity pattern or the associated biomechanical measures. AMAP scores demonstrate that at times more EMG activity may be counterproductive if it is not coming at the right phase of the gait cycle.

### Increase in BWS improves plantarflexor activity

**Fig. 4:**
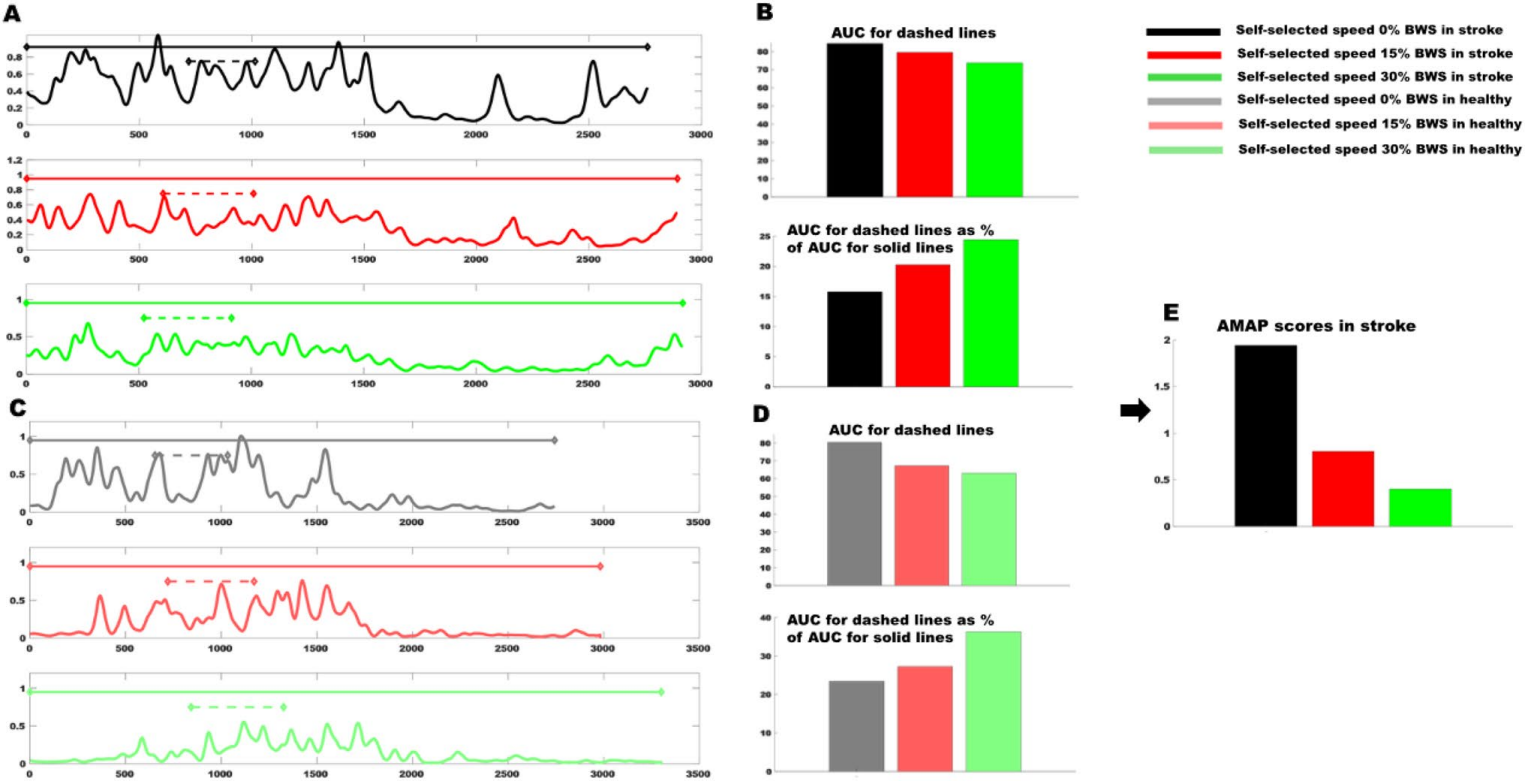
**A** Representative data of a stroke survivor: dashed lines represent first half of single-leg stance (SS1) and solid lines represent total gait cycle (GC) for Soleus activity. **B** Top panel: solid rectangles represent absolute EMG amplitude during SS1 and bottom panel: solid rectangles represent % of GC amplitude during SS1 for stroke participant. **C** Representative data of a healthy participant: dashed lines represent first half of single-leg stance (SS1) and solid lines represent total gait cycle (GC) for Soleus activity. **D** Top panel: solid rectangles represent absolute EMG amplitude during SS1 and bottom panel: solid rectangles represent % of GC amplitude during SS1 for healthy participant. Note that EMG amplitude during SS1, quantified as area under the curve (AUC) for dashed lines, decreases but % of GC amplitude during SS1 increases (i.e., AUC for dashed lines as % of AUC for solid lines). **E** Soleus AMAP amplitude scores for stroke participant improve with increased BWS. Scores closer to zero represent EMG pattern more similar to healthy controls walking at similar speeds and % BWS

We observed improved AMAP scores for plantarflexors (i.e., increased muscle activation pattern for SO and MG) during stance with increased BWS (i.e., decreased loading). Typically, one would expect diminished plantarflexor activity with increased BWS during stance phase (Hesse et al. 1997, 1999). However, in the current study we computed the EMG amplitude within each bin as percentage of the total EMG activity in gait cycle. Since, the plantarflexor activity during stance increases relative to the total gait cycle activity with increased BWS, plantarflexor activity appears to increase. For example, the SO peak activity during SS1 may appear reduced with increased BWS, but if “on” time during SS1 relative to the “on” time in a gait cycle increased, the EMG activity would be increased for that phase i.e., SS1 (Fig. 4). Our results suggest that this relative increase of SO activity during stance approaches the pattern of healthy controls at similar percent of BWS i.e., improved AMAP scores, whereas increased MG activity during DS1 is less similar to healthy patterns i.e., worse AMAP scores. These specific examples support our argument that AMAP provides a comprehensive understanding of changes in both timing and amplitude of muscle activity patterns by accounting for individual and biomechanical differences at different speeds and BWS.

### Trunk and feet assistance improves plantarflexor activity during stance

Therapist assistance at 30% BWS improved AMAP scores (i.e., decreased exaggerated plantarflexor activity observed at baseline) during early stance and late swing, and improved AMAP scores (i.e., increased diminished baseline plantartflexor activity) during mid-stance. Individuals with stroke often have an early activation of plantar flexors during stance along with decreased amplitude during mid and late stance (Knutsson and Richards 1979). We found that, 30% BWS and assistance to trunk and both feet improved scores (i.e., decreased the early activation of plantar flexors) during stance. Additionally, almost all therapist-assisted conditions resulted in improved amplitude and timing AMAP scores of SO and MG activity (i.e., increased) during mid and late stance. We believe, assistance to the trunk and both feet may promote appropriate limb loading at the desired time in the gait cycle, facilitating a more normal, reduced amplitude pattern of plantarflexors during early stance. Although, increased speed without therapist assistance resulted in worse timing and amplitude scores throughout stance, therapist assistance at fastest speeds improved AMAP scores. Therefore, training at faster speeds alone is not ideal to improve plantarflexor activity during stance. Our current results suggest therapist-assisted LT_SS_ at 30% BWS and self-selected walking speeds induced more appropriate muscle activation patterns compared to fast walking alone. Understanding the longer-term effects of repeated sessions of LT in these conditions is beyond the scope of the current study with this experimental paradigm.

### Non-paretic foot assistance improves plantarflexor activity during swing

At baseline self-selected speeds, individuals with stroke exhibited plantarflexor activity during the end of swing phase, which is typically negligible in healthy individuals. The altered pattern typically seen in individuals with stroke is due to abnormal merging (i.e. co-excitation) of plantarflexor activity with other stance phase muscle activity (McGowan et al. 2010; Brough et al. 2019). With increased BWS, we observed improved SO AMAP scores during late swing (i.e., decreased activity) that further improved with non-paretic foot assistance. Non-paretic foot assistance with 30% BWS may promote symmetric gait leading to a more normal co-excitation patterns of plantarflexors during swing.

### Trunk and feet assistance improves GM activity during stance but makes it worse during swing

BWS with assistance to trunk and paretic foot improved AMAP scores for GM (i.e., increased diminished baseline GM activity) during stance and increased BWS without therapist assistance improved AMAP score (i.e., decreased exaggerated baseline GM activity) during swing, but therapist assistance to trunk and both feet led to worse AMAP scores (i.e., increased GM activity) during swing. Diminished baseline GM activity seen in stroke gait relative to healthy controls during stance improved with increased BWS and therapist assistance to paretic foot, trunk, and trunk with paretic foot. We believe that therapist assistance to the trunk and paretic leg may promote appropriate weight bearing during stance phase facilitating increased GM activity during stance. At baseline, during swing we observed exaggerated GM activity in individuals with stroke. Consistent with previous literature (Hesse et al. 1997), this exaggerated baseline activity was improved with increased weight support. However, we observed worse AMAP scores for GM during swing with therapist assistance to trunk and both feet (i.e., further increased amplitude and longer “on” time). Although, not statistically significant we also observed worse AMAP scores during swing for all paretic foot assistance conditions. The goal of therapist assistance was to facilitate symmetric step length, which is the antero-posterior (AP) component of foot position. Lateral foot position control (Kao and Srivastava 2018) and increased GM activity during swing phase (Dean and Kautz 2015) are associated with dynamic stabilization during walking. While manipulating the AP foot position during swing, paretic foot assistance may have altered the ideal lateral foot position to maintain dynamic balance leading to increased GM activity as a compensatory mechanism for dynamic stabilization whereas, with increased BWS stroke survivors were likely better able to control their foot placement, leading to a more normal GM pattern. Therefore, LT_SS_ at 30% BWS without therapist assistance may be preferred to improve GM activity patterns during swing phase.

### Other muscles

BWS at faster speeds with therapist assistance to trunk and feet improved LH activity during stance, however TA activity patterns did not improve with any therapist-assisted LT_SS_ parameters. LH scores for most of the individuals with stroke were within normal variability both at baseline and following training. During early stance, LH amplitude AMAP scores improved at faster speeds and following therapist assistance, with most of the improvement seen for conditions that involve assistance to trunk and/or both feet. Therefore, it appears that assistance to trunk and/or both feet improve LH activity patterns during stance. Although for TA, scores during DS2 improved with 30% BWS (i.e., decrease in exaggerated baseline patterns), they became worse (i.e., increased) with assistance to non-paretic foot or trunk, and no changes were observed for the timing patterns with therapist assistance. Therefore, typically-used LT_SS_ parameters may not be ideal to improve altered TA activity in individuals with stroke. Activation patterns of MH, LH, RF, and VM in stroke survivors were similar to healthy controls at baseline and likely did not require large modifications to improve their patterns. Although this limits our ability to evaluate the effects of LT_SS_ parameters for these muscles, we have discussed in our previous work that SO, MG, and GM are typically more associated with functional walking ability of individuals with stroke (Hall et al. 2011; Srivastava et al. 2019) and therefore are more relevant for an appropriately targeted gait rehabilitation.

### Limitations and future directions

While the current study investigates the effects of various parameters during a single session of locomotor training, there are limitations that should be considered. Evaluating the influence of training parameters during LT_SS_ on muscle activity patterns using AMAP for gait rehabilitation was effective because AMAP has the ability to quantify differences in both EMG timing and amplitude observed during all subphases of the gait cycle (Srivastava et al. 2019). However, it is possible that a larger sample size of stroke survivors including more individuals with moderate to severe impairment levels is needed to generalize findings from the current study and aid clinicians in developing and evaluating a more personalized approach of long-term LT. Note that we envision AMAP as a tool for researchers to study how variations in LT_SS_ parameters affect and optimize EMG patterns rather than for clinicians to use it in developing protocols for individual people. Additionally, we did not investigate whether immediate changes in muscle activity patterns following exposure to LT_SS_ parameter variation are associated with biomechanical changes or functional walking recovery. Therefore, future studies are needed to understand the effects of LT parameterized to target specific muscle activation abnormalities on biomechanical changes with locomotor recovery following stroke. Future studies on evaluation of biomechanical changes and muscle activity patterns of the non-paretic leg in response to LT_SS_ parameter variation are also warranted.

Results from this study provide useful information on the effects of speed, level of BWS, and type of therapist assistance and their interaction during a single session of LT on changes in muscle activity patterns that may not be possible to capture by clinical or biomechanical measures. Armed with this information researchers can evaluate the effects of long-term LT training on specific biomechanical and clinical impairments to develop an effective rehabilitation approach. For example, future investigations can reveal whether individuals with impaired balance and altered foot placement strategy due to altered GM activity may benefit more from LT at 30% BWS without therapists’ assistance in comparison to LT with therapists’ assistance. Similarly, individuals with asymmetric gait and altered plantarflexor activity patterns may benefit more from LT at 30% BWS with therapists’ assistance at self-selected walking speeds in comparison to fast walking speeds.

## Conclusion

In conclusion, our results demonstrate that a specific set of LT_SS_ parameters can improve patterns of the amplitude and timing for SO, MG, and GM activity as quantified using the AMAP, but different sets of training parameters are needed depending on the muscles and gait sub-phases in which the specific changes in activation patterns are desired. LT_SS_ involving assistance to the trunk and feet that focuses on symmetric weight bearing and appropriate paretic limb loading improves activity of muscles involved in weight bearing during stance (i.e., SO, MG, and GM). Plantarflexor activity during swing improves with non-paretic foot assistance aimed at facilitating spatiotemporal gait symmetry. Additionally, LT_SS_ with increased BWS and not speed may result in a plantarflexor pattern more similar to healthy individuals suggesting that use of faster speeds for LT_SS_ should be considered in this light given the goals of the therapist. Based on the current results, we believe that optimally configured LT has the potential to improve muscle activity patterns which could lead to an improved walking ability in individuals with stroke.

## Supplementary Information

Below is the link to the electronic supplementary material.

Supplementary file1 (DOCX 44 kb)

Supplementary file2 (DOCX 51 kb)

Supplementary file3 (DOCX 46 kb)

## Data Availability

The data used in this study are available from the corresponding author upon reasonable request.

## Abbreviations

EMG: Electromyography
LT: Locomotor training
BWS: Body weight support
AMAP: Altered muscle activation patterns
TA: Tibialis anterior
SO: Soleus
MG: Medial gastrocnemius
VM: Vastus medialis
RF: Rectus femoris
LH: Lateral hamstring
MH: Medial hamstring
GM: Gluteus medius
GRF: Ground reaction force
DS1: First double support
SS1: First half of single-leg stance
SS2: Second half of single-leg stance
DS2: Second double support
SW1: First half of swing
SW2: Second half of swing
SD: Standard deviations
SS: Self-selected
P: Paretic foot assistance
N: Non-paretic foot assistance
T: Trunk assistance
PT: Paretic foot and trunk assistance
NT: Non-paretic foot assistance
PNT: Both feet and trunk assistance
PN: Both feet assistance
