# Supplementary material for: Variation of body weight supported treadmill training parameters during a single session can modulate muscle activity patterns in post-stroke gait
Source: Exp Brain Res. 2023 Jan 13;241(2):615–27. doi: 10.1007/s00221-023-06551-7 (PMC9895011; doi:10.1007/s00221-023-06551-7)
Supplement: Supplementary file 1 — Supplementary file1 (DOCX 44 kb) [file 221_2023_6551_MOESM1_ESM.docx]

Table 1. AMPLITUDE AND TIMING AMAP SCORES FOR INDIVIDUALS WITH STROKE WITHOUT THERAPIST ASSITANCE

| **SOLEUS** | | | | | | |  |
| --- | --- | --- | --- | --- | --- | --- | --- |
|  | **DS1** | **SS1** | **SS2** | **DS2** | **SW1** | **SW2** | |
| **Average and SD Total AMP Scores for Amplitude Component** | | | | | | |  |
| **SS_0% Mean±SD** | 2.49±1.31 | 1.51±1.09 | 2.17±1.29 | 0.83±0.49 | 1.33±2.23 | 4.22±4.28 | |
| **SS_15% Mean±SD** | 3.41±1.66 | 0.85±0.76 | 1.39±0.73 | 0.74±0.50 | 0.79±1.07 | 2.19±2.28 | |
| **FC_15% Mean±SD** | 3.47±1.65 | 1.00±0.74 | 1.58±0.69 | 0.93±0.59 | 0.78±0.85 | 2.65±2.59 | |
| **SS_30% Mean±SD** | 2.67±1.28 | 0.70±0.54 | 1.28±0.67 | 0.73±0.42 | 0.67±0.65 | 1.70±1.69 | |
| **FC_30% Mean±SD** | 2.81±2.07 | 0.90±0.64 | 1.37±0.77 | 0.92±0.53 | 0.63±0.85 | 1.70±1.74 | |
| **Average and SD Total AMP Scores for Timing Component** | | | | | | |  |
| **SS_0% Mean±SD** | 1.78±0.88 | 1.82±2.26 | 1.83±3.05 | 0.85±0.55 | 1.02±1.27 | 3.95±3.37 | |
| **SS_15% Mean±SD** | 2.07±1.02 | 0.91±0.83 | 1.12±1.57 | 0.88±0.49 | 0.66±0.69 | 1.89±1.53 | |
| **FC_15% Mean±SD** | 2.32±1.03 | 0.89±0.86 | 1.58±3.07 | 0.93±0.59 | 0.63±0.55 | 2.31±2.02 | |
| **SS_30% Mean±SD** | 1.69±0.99 | 0.96±0.89 | 1.17±1.58 | 0.86±0.56 | 0.61±0.39 | 1.56±1.31 | |
| **FC_30% Mean±SD** | 1.83±0.94 | 0.92±0.80 | 1.38±2.22 | 0.95±0.64 | 0.65±0.78 | 1.85±1.71 | |

| **MEDIAL GASTROCNEMIUS** | | | | | | |  |
| --- | --- | --- | --- | --- | --- | --- | --- |
|  | **DS1** | **SS1** | **SS2** | **DS2** | **SW1** | **SW2** | |
| **Average and SD Total AMP Scores for Amplitude Component** | | | | | | |  |
| **SS_0% Mean±SD** | 2.40±1.66 | 1.15±0.61 | 1.59±0.68 | 0.93±0.57 | 0.84±0.76 | 3.83±2.65 | |
| **SS_15% Mean±SD** | 3.22±2.16 | 0.71±0.56 | 1.46±0.79 | 0.71±0.54 | 0.69±0.34 | 2.36±2.18 | |
| **FC_15% Mean±SD** | 3.84±2.24 | 0.72±0.43 | 1.88±0.72 | 0.59±0.51 | 0.59±0.37 | 2.64±2.21 | |
| **SS_30% Mean±SD** | 3.45±2.34 | 0.60±0.46 | 1.64±0.70 | 0.54±0.40 | 0.59±0.43 | 2.56±2.43 | |
| **FC_30% Mean±SD** | 3.75±2.39 | 0.61±0.36 | 1.80±0.82 | 0.45±0.35 | 0.56±0.39 | 2.42±1.99 | |
| **Average and SD Total AMP Scores for Timing Component** | | | | | | |  |
| **SS_0% Mean±SD** | 2.20±1.19 | 0.88±0.64 | 1.47±1.59 | 1.25±.92 | 0.74±0.65 | 3.05±1.90 | |
| **SS_15% Mean±SD** | 2.25±1.12 | 0.71±0.45 | 0.99±1.20 | 0.95±0.66 | 0.67±0.43 | 1.85±1.33 | |
| **FC_15% Mean±SD** | 2.52±1.01 | 0.71±0.44 | 1.46±2.05 | 1.08±0.81 | 0.60±0.45 | 1.96±1.27 | |
| **SS_30% Mean±SD** | 2.34±1.26 | 0.76±0.39 | 0.90±0.90 | 0.98±0.74 | 0.68±0.45 | 2.15±1.64 | |
| **FC_30% Mean±SD** | 2.51±1.33 | 0.82±0.29 | 1.00±1.26 | 0.88±0.64 | 0.60±0.36 | 2.14±1.58 | |

| **MEDIAL HAMSTRINGS** | | | | | | |  |
| --- | --- | --- | --- | --- | --- | --- | --- |
|  | **DS1** | **SS1** | **SS2** | **DS2** | **SW1** | **SW2** | |
| **Average and SD Total AMP Scores for Amplitude Component** | | | | | | |  |
| **SS_0% Mean±SD** | 0.60±0.41 | 0.57±0.33 | 0.71±0.51 | 0.86±0.89 | 0.78±0.85 | 0.60±0.46 | |
| **SS_15% Mean±SD** | 0.64±0.52 | 0.54±0.35 | 0.58±0.39 | 1.09±1.09 | 0.67±0.95 | 0.84±0.49 | |
| **FC_15% Mean±SD** | 0.69±0.54 | 0.48±0.31 | 0.60±0.32 | 1.20±0.85 | 0.79±1.12 | 0.77±0.49 | |
| **SS_30% Mean±SD** | 0.62±0.46 | 0.43±0.29 | 0.44±0.27 | 1.02±0.95 | 0.65±0.77 | 0.57±0.40 | |
| **FC_30% Mean±SD** | 0.71±0.56 | 0.39±0.27 | 0.50±0.32 | 0.94±0.91 | 0.90±1.14 | 0.55±0.51 | |
| **Average and SD Total AMP Scores for Timing Component** | | | | | | |  |
| **SS_0% Mean±SD** | 0.71±0.45 | 0.91±0.59 | 1.17±0.82 | 1.10±0.80 | 0.91±0.52 | 0.64±0.40 | |
| **SS_15% Mean±SD** | 0.77±0.62 | 0.70±0.44 | 1.15±0.88 | 1.24±1.00 | 0.77±0.58 | 0.77±0.45 | |
| **FC_15% Mean±SD** | 0.78±0.57 | 0.83±0.56 | 1.15±0.79 | 1.40±0.79 | 0.84±0.59 | 0.81±0.65 | |
| **SS_30% Mean±SD** | 0.58±0.50 | 0.62±0.39 | 0.72±0.51 | 1.10±0.87 | 0.96±0.66 | 0.96±0.46 | |
| **FC_30% Mean±SD** | 0.57±0.48 | 0.75±0.45 | 0.75±0.60 | 1.05±0.73 | 0.94±0.73 | 0.87±0.45 | |

| **LATERAL HAMSTRINGS** | | | | | | |  |
| --- | --- | --- | --- | --- | --- | --- | --- |
|  | **DS1** | **SS1** | **SS2** | **DS2** | **SW1** | **SW2** | |
| **Average and SD Total AMP Scores for Amplitude Component** | | | | | | |  |
| **SS_0% Mean±SD** | 0.93±0.59 | 1.00±0.64 | 1.01±0.87 | 0.92±0.48 | 0.88±0.43 | 0.80±0.72 | |
| **SS_15% Mean±SD** | 1.14±0.73 | 0.79±0.63 | 0.77±0.50 | 0.83±0.43 | 1.09±0.61 | 0.78±0.78 | |
| **FC_15% Mean±SD** | 0.92±0.72 | 1.04±0.80 | 0.90±0.72 | 0.89±0.43 | 1.03±0.40 | 0.64±0.48 | |
| **SS_30% Mean±SD** | 1.08±0.66 | 0.88±0.72 | 0.80±0.65 | 0.88±0.43 | 0.87±0.29 | 0.77±0.66 | |
| **FC_30% Mean±SD** | 0.92±0.64 | 1.17±0.84 | 0.73±0.65 | 0.88±0.47 | 0.84±0.29 | 0.61±0.60 | |
| **Average and SD Total AMP Scores for Timing Component** | | | | | | |  |
| **SS_0% Mean±SD** | 1.28±0.39 | 1.66±0.57 | 1.39±0.97 | 1.09±0.63 | 0.96±0.56 | 0.97±0.66 | |
| **SS_15% Mean±SD** | 1.22±0.33 | 1.57±0.60 | 1.33±1.00 | 0.90±0.59 | 1.11±0.83 | 0.95±0.70 | |
| **FC_15% Mean±SD** | 1.19±0.40 | 1.72±0.62 | 1.29±0.95 | 1.02±0.53 | 1.18±0.78 | 0.82±0.66 | |
| **SS_30% Mean±SD** | 1.20±0.32 | 1.50±0.72 | 1.12±0.89 | 0.99±0.46 | 0.98±0.66 | 1.10±0.75 | |
| **FC_30% Mean±SD** | 1.18±0.35 | 1.72±0.69 | 0.95±0.83 | 0.98±0.48 | 0.94±0.66 | 0.92±0.67 | |

| **TIBIALIS ANTERIOR** | | | | | | |  |
| --- | --- | --- | --- | --- | --- | --- | --- |
|  | **DS1** | **SS1** | **SS2** | **DS2** | **SW1** | **SW2** | |
| **Average and SD Total AMP Scores for Amplitude Component** | | | | | | |  |
| **SS_0% Mean±SD** | 1.10±0.68 | 1.06±1.00 | 0.89±0.78 | 2.75±1.76 | 1.09±0.94 | 1.17±0.65 | |
| **SS_15% Mean±SD** | 1.12±0.88 | 1.03±1.04 | 0.73±0.65 | 2.73±1.95 | 1.13±1.01 | 1.09±0.77 | |
| **FC_15% Mean±SD** | 1.50±1.01 | 1.02±1.24 | 0.67±0.53 | 3.11±2.09 | 1.33±1.16 | 1.18±0.84 | |
| **SS_30% Mean±SD** | 1.16±0.81 | 0.87±1.17 | 0.69±0.56 | 3.26±2.80 | 0.93±0.74 | 1.07±0.75 | |
| **FC_30% Mean±SD** | 1.36±0.83 | 0.83±0.46 | 0.60±0.50 | 3.87±2.85 | 1.09±0.87 | 1.18±0.80 | |
| **Average and SD Total AMP Scores for Timing Component** | | | | | | |  |
| **SS_0% Mean±SD** | 1.03±0.73 | 0.99±0.60 | 0.93±0.58 | 2.93±1.63 | 1.09±0.96 | 1.37±0.96 | |
| **SS_15% Mean±SD** | 1.27±0.91 | 1.07±0.66 | 0.85±0.56 | 2.64±1.76 | 1.12±0.88 | 1.31±0.86 | |
| **FC_15% Mean±SD** | 1.29±1.00 | 1.10±0.73 | 0.82±0.51 | 2.82±1.63 | 1.42±1.26 | 1.46±0.93 | |
| **SS_30% Mean±SD** | 1.33±0.85 | 0.92±0.68 | 0.78±0.42 | 2.18±1.63 | 1.02±0.80 | 1.21±0.79 | |
| **FC_30% Mean±SD** | 1.37±0.85 | 1.02±0.63 | 0.71±0.44 | 2.35±1.71 | 1.14±0.91 | 1.30±0.88 | |

| **RECTUS FEMORIS** | | | | | | |  |
| --- | --- | --- | --- | --- | --- | --- | --- |
|  | **DS1** | **SS1** | **SS2** | **DS2** | **SW1** | **SW2** | |
| **Average and SD Total AMP Scores for Amplitude Component** | | | | | | |  |
| **SS_0% Mean±SD** | 0.77±0.43 | 1.21±0.82 | 0.83±0.58 | 1.42±0.88 | 0.80±0.50 | 1.12±0.99 | |
| **SS_15% Mean±SD** | 0.77±0.60 | 0.78±0.64 | 0.67±0.49 | 1.36±0.88 | 0.69±0.27 | 1.02±1.13 | |
| **FC_15% Mean±SD** | 0.74±0.64 | 0.85±0.62 | 0.72±0.58 | 1.51±1.02 | 0.63±0.35 | 1.00±1.18 | |
| **SS_30% Mean±SD** | 0.79±0.49 | 0.73±0.52 | 0.99±0.49 | 1.25±0.83 | 0.74±0.28 | 1.05±1.15 | |
| **FC_30% Mean±SD** | 0.92±0.48 | 0.82±0.50 | 0.96±0.57 | 1.33±0.90 | 0.79±0.59 | 1.24±1.16 | |
| **Average and SD Total AMP Scores for Timing Component** | | | | | | |  |
| **SS_0% Mean±SD** | 0.87±0.69 | 1.12±0.73 | 0.95±0.55 | 1.37±0.83 | 0.79±0.51 | 1.04±0.76 | |
| **SS_15% Mean±SD** | 1.44±1.57 | 1.06±0.62 | 1.01±0.51 | 1.32±0.83 | 0.69±0.27 | 1.24±0.81 | |
| **FC_15% Mean±SD** | 1.00±1.56 | 0.91±0.69 | 0.95±0.59 | 1.26±0.97 | 0.63±0.35 | 1.03±0.62 | |
| **SS_30% Mean±SD** | 0.83±0.68 | 0.93±0.48 | 1.31±0.77 | 1.31±0.87 | 0.74±0.28 | 1.14±0.79 | |
| **FC_30% Mean±SD** | 0.79±0.94 | 1.00±0.55 | 1.32±0.85 | 1.25±0.85 | 0.79±0.59 | 1.35±0.66 | |

| **VASTUS MEDIALIS** | | | | | | |  |
| --- | --- | --- | --- | --- | --- | --- | --- |
|  | **DS1** | **SS1** | **SS2** | **DS2** | **SW1** | **SW2** | |
| **Average and SD Total AMP Scores for Amplitude Component** | | | | | | |  |
| **SS_0% Mean±SD** | 0.47±0.28 | 0.91±0.69 | 0.99±0.90 | 0.57±0.28 | 0.60±0.21 | 1.09±0.91 | |
| **SS_15% Mean±SD** | 0.49±0.36 | 0.74±0.54 | 0.79±0.61 | 0.49±0.34 | 0.61±0.22 | 1.08±1.00 | |
| **FC_15% Mean±SD** | 0.59±0.44 | 0.83±0.63 | 0.87±0.64 | 0.52±0.35 | 0.63±0.31 | 0.97±0.92 | |
| **SS_30% Mean±SD** | 0.51±0.47 | 0.87±0.62 | 1.36±1.15 | 0.52±0.35 | 0.72±0.22 | 0.96±0.86 | |
| **FC_30% Mean±SD** | 0.74±0.59 | 0.99±0.62 | 1.76±1.44 | 0.56±0.39 | 0.76±0.43 | 1.15±0.92 | |
| **Average and SD Total AMP Scores for Timing Component** | | | | | | |  |
| **SS_0% Mean±SD** | 0.92±0.39 | 1.42±0.50 | 1.50±1.08 | 0.64±0.39 | 0.63±0.22 | 0.95±0.72 | |
| **SS_15% Mean±SD** | 0.78±0.28 | 1.30±0.54 | 1.39±1.12 | 0.66±0.48 | 0.69±0.27 | 1.01±0.68 | |
| **FC_15% Mean±SD** | 0.72±0.30 | 1.40±0.64 | 1.71±1.18 | 0.77±0.44 | 0.63±0.35 | 0.89±0.68 | |
| **SS_30% Mean±SD** | 0.81±0.23 | 1.39±0.67 | 1.59±1.22 | 0.84±0.57 | 0.74±0.28 | 0.91±0.63 | |
| **FC_30% Mean±SD** | 0.89±0.75 | 1.43±0.77 | 1.81±1.24 | 0.85±0.54 | 0.79±0.59 | 0.99±0.63 | |

| **GLUTEUS MEDIUS** | | | | | | |  |
| --- | --- | --- | --- | --- | --- | --- | --- |
|  | **DS1** | **SS1** | **SS2** | **DS2** | **SW1** | **SW2** | |
| **Average and SD Total AMP Scores for Amplitude Component** | | | | | | |  |
| **SS_0% Mean±SD** | 0.98±0.77 | 2.04±1.34 | 1.32±0.63 | 1.82±1.70 | 1.01±0.85 | 1.54±1.39 | |
| **SS_15% Mean±SD** | 0.87±0.70 | 1.61±1.09 | 1.13±0.71 | 1.60±1.55 | 0.74±0.48 | 1.43±1.11 | |
| **FC_15% Mean±SD** | 0.85±0.55 | 1.63±1.09 | 0.84±0.66 | 1.93±1.53 | 0.95±0.90 | 1.48±1.20 | |
| **SS_30% Mean±SD** | 0.72±0.55 | 0.93±0.56 | 0.94±0.70 | 0.88±0.68 | 0.67±0.52 | 1.08±0.95 | |
| **FC_30% Mean±SD** | 0.69±0.46 | 1.02±0.77 | 0.78±0.53 | 1.06±0.88 | 0.66±0.52 | 1.53±1.20 | |
| **Average and SD Total AMP Scores for Timing Component** | | | | | | |  |
| **SS_0% Mean±SD** | 1.01±1.04 | 2.35±3.75 | 1.14±0.78 | 1.43±1.06 | 0.85±0.64 | 1.34±1.09 | |
| **SS_15% Mean±SD** | 1.03±0.55 | 2.65±4.46 | 1.18±1.00 | 1.31±1.036 | 0.65±0.48 | 1.33±0.96 | |
| **FC_15% Mean±SD** | 0.90±0.58 | 3.05±4.68 | 0.74±0.72 | 1.52±1.02 | 0.79±0.42 | 1.27±0.99 | |
| **SS_30% Mean±SD** | 1.01±0.66 | 0.54±0.36 | 0.86±0.53 | 1.03±0.77 | 0.70±0.45 | 1.02±0.88 | |
| **FC_30% Mean±SD** | 0.85±0.56 | 0.77±0.97 | 0.80±0.46 | 0.99±0.84 | 0.75±0.49 | 1.15±0.99 | |
