# Supplementary material for: Variation of body weight supported treadmill training parameters during a single session can modulate muscle activity patterns in post-stroke gait
Source: Exp Brain Res. 2023 Jan 13;241(2):615–27. doi: 10.1007/s00221-023-06551-7 (PMC9895011; doi:10.1007/s00221-023-06551-7)
Supplement: Supplementary file 2 — Supplementary file2 (DOCX 51 kb) [file 221_2023_6551_MOESM2_ESM.docx]

Table 2. AMPLITUDE AND TIMING AMAP SCORES FOR INDIVIDUALS WITH STROKE WITH THERAPIST ASSISTANCE

| **SOLEUS** | | | | | | |  |
| --- | --- | --- | --- | --- | --- | --- | --- |
|  | **DS1** | **SS1** | **SS2** | **DS2** | **SW1** | **SW2** | |
| **Average and SD Total AMP Scores for Amplitude Component** | | | | | | |  |
| **SS Mean±SD** | 2.49±1.31 | 1.51±1.09 | 2.17±1.29 | 0.83±0.49 | 1.33±2.23 | 4.22±4.28 | |
| **PF Mean±SD** | 2.05±1.37 | 0.87±0.84٭ | 1.50±1.13٭ | 0.72±0.50 | 0.96±1.42 | 3.52±3.60 | |
| **NF Mean±SD** | 2.24±1.31 | 0.81±0.69٭ | 1.37±1.09٭ | 0.91±0.49 | 1.13±1.77 | 2.29±3.07٭ | |
| **Tr Mean±SD** | 2.49±1.48 | 0.82±0.71٭ | 1.48±1.05٭ | 0.96±0.51 | 1.13±1.65 | 2.58±3.60 | |
| **Tr_PF Mean±SD** | 1.94±1.32 | 0.80±0.73٭ | 1.41±1.05٭ | 0.79±0.47 | 1.05±1.71 | 3.11±3.56 | |
| **Tr_NF Mean±SD** | 2.17±0.98 | 0.73±0.67٭ | 1.31±1.06٭ | 1.00±0.58 | 1.13±1.54 | 2.79±3.37 | |
| **Tr_PF_NF Mean±SD** | 1.51±0.95٭ | 0.85±0.64٭ | 1.20±1.09٭ | 0.82±0.44 | 1.04±1.51 | 3.07±4.00 | |
| **PF_NF Mean±SD** | 1.74±1.25 | 0.86±0.71٭ | 1.19±1.12٭ | 0.82±0.45 | 0.90±1.57 | 2.84±3.92 | |
| **Average and SD Total AMP Scores for Timing Component** | | | | | | |  |
| **SS Mean±SD** | 1.78±0.88 | 1.82±2.26 | 1.83±3.05 | 0.85±0.55 | 1.02±1.27 | 3.95±3.37 | |
| **PF Mean±SD** | 2.10±0.92 | 0.97±0.96٭ | 2.18±3.69 | 0.76±0.59 | 0.90±1.04 | 3.19±2.56 | |
| **NF Mean±SD** | 1.82±1.00 | 0.94±0.80٭ | 2.13±3.32 | 0.83±0.58 | 0.88±1.04 | 2.24±2.10٭ | |
| **Tr Mean±SD** | 1.91±0.90 | 1.08±0.87 | 2.20±3.57 | 1.00±0.61 | 0.90±1.16 | 2.48±2.39٭ | |
| **Tr_PF Mean±SD** | 1.98±0.88 | 0.88±0.78 | 2.07±3.57 | 0.79±0.59 | 0.88±1.08 | 3.15±2.54 | |
| **Tr_NF Mean±SD** | 1.87±0.91 | 1.01±0.74 | 2.15±3.42 | 1.02±0.63 | 0.98±1.03 | 2.68±2.20 | |
| **Tr_PF_NF Mean±SD** | 1.85±0.84 | 0.90±0.90 | 2.05±3.55 | 0.81±0.55 | 0.86±0.97 | 2.85±2.60 | |
| **PF_NF Mean±SD** | 1.81±1.02 | 1.07±0.93 | 2.03±3.55 | 0.85±0.51 | 0.84±1.13 | 2.83±2.62 | |

| **MEDIAL GASTROCNEMIUS** | | | | | | |  |
| --- | --- | --- | --- | --- | --- | --- | --- |
|  | **DS1** | **SS1** | **SS2** | **DS2** | **SW1** | **SW2** | |
| **Average and SD Total AMP Scores for Amplitude Component** | | | | | | |  |
| **SS Mean±SD** | 2.40±1.66 | 1.15±0.61 | 1.59±0.68 | 0.93±0.57 | 0.84±0.76 | 3.83±2.65 | |
| **PF Mean±SD** | 2.65±1.44 | 0.72±0.45 | 1.91±0.74 | 0.53±0.52 | 1.02±1.12 | 5.03±3.07 | |
| **NF Mean±SD** | 3.12±1.86 | 0.53±0.46٭ | 1.76±0.74 | 0.55±0.42٭ | 0.70±0.57 | 2.95±2.54 | |
| **Tr Mean±SD** | 3.19±1.81 | 0.60±0.49٭ | 1.90±0.83 | 0.55±0.35٭ | 0.80±0.80 | 3.58±2.80 | |
| **Tr_PF Mean±SD** | 2.48±1.75 | 0.74±0.53 | 1.87±0.76 | 0.49±0.45٭ | 0.87±0.89 | 4.99±3.33 | |
| **Tr_NF Mean±SD** | 2.98±1.40 | 0.50±0.36٭ | 1.82±0.72 | 0.53±0.34٭ | 0.67±0.54 | 3.28±2.45 | |
| **Tr_PF_NF Mean±SD** | 2.00±1.14 | 0.62±0.46٭ | 1.65±0.71 | 0.51±0.43٭ | 0.86±0.76 | 4.56±2.29 | |
| **PF_NF Mean±SD** | 2.30±1.36 | 0.62±0.51 | 1.79±.074 | 0.62±0.69 | 0.96±0.90 | 4.32±2.30 | |
| **Average and SD Total AMP Scores for Timing Component** | | | | | | |  |
| **SS Mean±SD** | 2.20±1.19 | 0.88±0.64 | 1.47±1.59 | 1.25±.92 | 0.74±0.65 | 3.05±1.90 | |
| **PF Mean±SD** | 2.79±1.26٭ | 0.60±0.48 | 1.14±1.20 | 1.11±0.95 | 0.92±0.88 | 3.96±2.21 | |
| **NF Mean±SD** | 2.57±1.14 | 0.65±0.38 | 1.01±0.88 | 1.16±0.98 | 0.71±0.55 | 2.79±2.05 | |
| **Tr Mean±SD** | 2.71±1.39 | 0.77±0.41 | 1.06±1.21 | 1.06±0.69 | 0.82±0.73 | 3.30±2.39 | |
| **Tr_PF Mean±SD** | 2.67±1.34 | 0.57±0.52 | 1.13±1.13 | 1.19±0.73 | 0.92±0.78 | 4.19±2.54 | |
| **Tr_NF Mean±SD** | 2.60±1.13 | 0.76±0.38 | 1.19±1.13 | 1.09±0.89 | 0.70±0.48 | 3.06±2.02 | |
| **Tr_PF_NF Mean±SD** | 1.27±1.09 | 0.64±0.48 | 1.27±1.09 | 1.14±0.79 | 0.90±0.73 | 3.98±2.22 | |
| **PF_NF Mean±SD** | 2.55±1.36 | 0.60±0.43 | 1.32±1.07 | 1.07±0.90 | 0.94±0.78 | 3.89±1.95 | |

| **MEDIAL HAMSTRINGS** | | | | | | |  |
| --- | --- | --- | --- | --- | --- | --- | --- |
|  | **DS1** | **SS1** | **SS2** | **DS2** | **SW1** | **SW2** | |
| **Average and SD Total AMP Scores for Amplitude Component** | | | | | | |  |
| **SS Mean±SD** | 0.60±0.41 | 0.57±0.33 | 0.71±0.51 | 0.86±0.89 | 0.78±0.85 | 0.60±0.46 | |
| **PF Mean±SD** | 0.87±0.47٭ | 0.58±0.30 | 0.56±0.41 | 0.84±0.77 | 1.35±1.02 | 0.77±0.62 | |
| **NF Mean±SD** | 0.73±0.47 | 0.62±0.40 | 0.62±0.58 | 1.13±0.95 | 0.83±0.99 | 0.51±0.32 | |
| **Tr Mean±SD** | 0.71±0.51 | 0.57±0.34 | 0.48±0.26 | 1.03±0.88 | 0.93±1.01 | 0.64±0.61 | |
| **Tr_PF Mean±SD** | 0.83±0.50٭ | 0.55±0.32 | 0.55±0.27 | 0.92±0.70 | 1.25±0.78 | 0.74±0.59 | |
| **Tr_NF Mean±SD** | 0.74±0.46 | 0.63±0.53 | 0.55±0.37 | 1.14±0.90 | 0.97±0.91 | 0.53±0.37 | |
| **Tr_PF_NF Mean±SD** | 0.86±0.46٭ | 0.46±0.32 | 0.63±0.29 | 0.74±0.57 | 1.22±1.00 | 0.63±0.48 | |
| **PF_NF Mean±SD** | 0.84±0.42٭ | 0.55±0.26 | 0.63±0.51 | 1.04±1.32 | 1.21±1.01 | 0.59±0.43 | |
| **Average and SD Total AMP Scores for Timing Component** | | | | | | |  |
| **SS Mean±SD** | 0.71±0.45 | 0.91±0.59 | 1.17±0.82 | 0.86±0.89 | 0.91±0.52 | 0.64±0.40 | |
| **PF Mean±SD** | 0.52±0.52 | 0.69±0.41 | 0.65±0.57 | 0.84±0.77 | 1.19±0.78 | 1.09±0.42٭ | |
| **NF Mean±SD** | 0.64±0.49 | 0.77±0.45 | 0.78±0.49 | 1.13±0.95 | 0.94±0.67 | 0.65±0.44 | |
| **Tr Mean±SD** | 0.67±0.52 | 0.67±0.55 | 0.72±0.41 | 1.03±0.88 | 1.03±0.71 | 0.75±0.43 | |
| **Tr_PF Mean±SD** | 0.71±0.46 | 0.67±0.42 | 0.72±0.39 | 0.92±0.70 | 1.30±0.75 | 1.13±0.36٭ | |
| **Tr_NF Mean±SD** | 0.54±0.46 | 0.78±0.44 | 0.72±0.49٭ | 1.14±0.90 | 1.17±0.71 | 0.76±0.44 | |
| **Tr_PF_NF Mean±SD** | 0.63±0.46 | 0.71±0.31 | 0.78±0.53 | 0.74±0.57 | 1.26±0.79 | 1.20±0.59٭ | |
| **PF_NF Mean±SD** | 0.60±0.46 | 0.61±0.37 | 0.71±0.54 | 1.04±1.32 | 1.21±0.83 | 1.08±0.35٭ | |

| **LATERAL HAMSTRINGS** | | | | | | |  |
| --- | --- | --- | --- | --- | --- | --- | --- |
|  | **DS1** | **SS1** | **SS2** | **DS2** | **SW1** | **SW2** | |
| **Average and SD Total AMP Scores for Amplitude Component** | | | | | | |  |
| **SS Mean±SD** | 0.93±0.59 | 1.00±0.64 | 1.01±0.87 | 0.92±0.48 | 0.88±0.43 | 0.80±0.72 | |
| **PF Mean±SD** | 0.48±0.46٭ | 0.98±0.62 | 0.94±0.90 | 0.93±0.43 | 0.60±0.25٭ | 0.64±0.52 | |
| **NF Mean±SD** | 0.70±0.45 | 1.49±0.93 | 0.79±0.75 | 0.86±0.53 | 0.81±0.31 | 0.48±0.36 | |
| **Tr Mean±SD** | 0.74±0.49 | 1.07±0.77 | 0.70±0.63 | 0.88±0.38 | 0.79±0.37 | 0.50±0.32 | |
| **Tr_PF Mean±SD** | 0.55±0.43 | 0.86±0.67 | 0.56±0.50 | 0.95±0.36 | 0.61±0.33٭ | 0.71±0.61 | |
| **Tr_NF Mean±SD** | 0.65±0.47 | 1.28±0.84 | 0.81±0.76 | 0.84±0.41 | 0.78±0.33٭ | 0.48±0.39 | |
| **Tr_PF_NF Mean±SD** | 0.45±0.41٭ | 1.18±0.74 | 0.76±0.61 | 0.95±0.38 | 0.61±0.28٭ | 0.56±0.50 | |
| **PF_NF Mean±SD** | 0.48±0.38٭ | 1.31±0.90 | 1.00±0.86 | 1.05±0.65 | 0.66±0.32٭ | 0.53±0.41 | |
| **Average and SD Total AMP Scores for Timing Component** | | | | | | |  |
| **SS Mean±SD** | 1.28±0.39 | 1.66±0.57 | 1.39±0.97 | 1.09±0.63 | 0.96±0.56 | 0.97±0.66 | |
| **PF Mean±SD** | 1.11±0.37 | 1.56±0.83 | 0.98±0.83 | 1.00±0.44 | 0.62±0.46٭ | 1.16±0.57 | |
| **NF Mean±SD** | 1.17±0.35 | 1.72±0.79 | 1.02±0.96 | 0.82±0.56 | 0.88±0.59 | 0.87±0.60 | |
| **Tr Mean±SD** | 1.10±0.40٭ | 1.61±0.69 | 0.84±0.78 | 0.89±0.48 | 0.97±0.59 | 0.90±0.58 | |
| **Tr_PF Mean±SD** | 1.16±0.37 | 1.46±0.76 | 0.79±0.65٭ | 0.92±0.53 | 0.76±0.49 | 1.29±0.60 | |
| **Tr_NF Mean±SD** | 1.02±0.45٭ | 1.50±0.70 | 1.01±0.77 | 0.97±0.50 | 0.91±0.50 | 0.98±0.57 | |
| **Tr_PF_NF Mean±SD** | 1.13±0.40 | 1.45±0.75 | 0.91±0.75 | 0.90±0.51 | 0.66±0.51 | 1.34±0.52٭ | |
| **PF_NF Mean±SD** | 1.06±0.33 | 1.66±0.79 | 1.04±0.84 | 1.02±0.51 | 0.72±0.52 | 1.28±0.52 | |

| **TIBIALIS ANTERIOR** | | | | | | |  |
| --- | --- | --- | --- | --- | --- | --- | --- |
|  | **DS1** | **SS1** | **SS2** | **DS2** | **SW1** | **SW2** | |
| **Average and SD Total AMP Scores for Amplitude Component** | | | | | | |  |
| **SS Mean±SD** | 1.10±0.68 | 1.06±1.00 | 0.89±0.78 | 2.75±1.76 | 1.09±0.94 | 1.17±0.65 | |
| **PF Mean±SD** | 1.01±0.64 | 1.44±1.51 | 1.12±1.21 | 3.33±2.46 | 1.33±0.76 | 1.37±0.83 | |
| **NF Mean±SD** | 1.33±0.81 | 1.29±1.44 | 1.01±1.14 | 3.84±2.35٭ | 1.24±0.98 | 1.38±0.85 | |
| **Tr Mean±SD** | 1.34±0.78 | 1.20±1.37 | 0.87±0.62 | 4.23±2.87٭ | 1.06±0.88 | 1.37±0.82 | |
| **Tr_PF Mean±SD** | 0.92±0.79 | 1.30±1.26 | 1.02±1.08 | 3.51±2.86 | 1.16±0.85 | 1.32±0.85 | |
| **Tr_NF Mean±SD** | 1.22±0.77 | 1.32±1.49 | 1.00±0.92 | 3.90±2.69 | 1.15±0.79 | 1.37±0.82 | |
| **Tr_PF_NF Mean±SD** | 0.88±0.72 | 1.83±1.62٭ | 1.19±1.29 | 3.21±2.68 | 1.19±0.87 | 1.31±0.83 | |
| **PF_NF Mean±SD** | 0.91±0.67 | 1.56±1.68 | 1.22±1.23 | 3.66±2.79 | 1.38±0.88 | 1.39±0.95 | |
| **Average and SD Total AMP Scores for Timing Component** | | | | | | |  |
| **SS Mean±SD** | 1.03±0.73 | 0.99±0.60 | 0.93±0.58 | 2.93±1.63 | 1.09±0.96 | 1.37±0.96 | |
| **PF Mean±SD** | 1.21±0.66 | 1.22±0.79 | 0.90±0.63 | 2.64±1.43 | 1.66±1.25 | 1.44±0.98 | |
| **NF Mean±SD** | 1.39±0.97 | 1.12±0.83 | 0.89±0.58 | 2.58±1.60 | 0.93±0.72 | 1.50±0.93 | |
| **Tr Mean±SD** | 1.31±0.85 | 1.13±0.86 | 0.84±0.46 | 2.38±1.63 | 1.21±1.15 | 1.52±0.88 | |
| **Tr_PF Mean±SD** | 1.11±0.84 | 1.26±0.88 | 0.93±0.67 | 2.57±1.59 | 1.56±1.28 | 1.34±1.05 | |
| **Tr_NF Mean±SD** | 1.33±0.94 | 1.15±0.84 | 0.92±0.63 | 2.63±1.66 | 1.27±0.95 | 1.42±0.94 | |
| **Tr_PF_NF Mean±SD** | 1.29±0.79 | 1.34±0.89 | 0.90±0.65 | 2.46±1.61 | 1.67±1.29 | 1.33±0.98 | |
| **PF_NF Mean±SD** | 0.91±0.67 | 1.19±0.93 | 0.92±0.65 | 2.46±1.49 | 1.73±1.30 | 1.63±0.92 | |

| **RECTUS FEMORIS** | | | | | | |  |
| --- | --- | --- | --- | --- | --- | --- | --- |
|  | **DS1** | **SS1** | **SS2** | **DS2** | **SW1** | **SW2** | |
| **Average and SD Total AMP Scores for Amplitude Component** | | | | | | |  |
| **SS Mean±SD** | 0.77±0.43 | 1.21±0.82 | 0.83±0.58 | 1.42±0.88 | 0.80±0.50 | 1.12±0.99 | |
| **PF Mean±SD** | 0.88±0.47 | 0.68±0.54 | 1.10±0.79 | 1.22±0.92 | 0.78±0.54 | 1.24±1.22 | |
| **NF Mean±SD** | 1.03±0.52 | 0.73±0.44 | 1.23±0.83 | 1.24±0.99 | 0.98±1.22 | 1.05±1.06 | |
| **Tr Mean±SD** | 0.86±0.56 | 0.76±0.50 | 1.11±0.75 | 1.24±0.84 | 0.93±0.91 | 1.16±1.23 | |
| **Tr_PF Mean±SD** | 0.84±0.44 | 0.63±0.47 | 1.00±0.71 | 1.35±0.94 | 0.78±0.51 | 1.18±1.12 | |
| **Tr_NF Mean±SD** | 0.98±0.55 | 0.82±0.57 | 1.25±0.93 | 1.28±0.83 | 0.90±0.74 | 1.13±1.04 | |
| **Tr_PF_NF Mean±SD** | 1.02±0.43 | 0.71±0.50 | 1.35±0.81 | 1.26±0.75 | 0.98±0.67 | 1.28±1.08 | |
| **PF_NF Mean±SD** | 1.02±0.48 | 0.67±0.51 | 1.36±0.68 | 1.24±1.27 | 0.90±0.62 | 1.23±1.09 | |
| **Average and SD Total AMP Scores for Timing Component** | | | | | | |  |
| **SS Mean±SD** | 0.87±0.69 | 1.12±0.73 | 0.95±0.55 | 1.37±0.83 | 0.79±0.51 | 1.04±0.76 | |
| **PF Mean±SD** | 0.62±0.46 | 1.03±0.45 | 1.46±1.03 | 1.41±0.78 | 1.00±0.56 | 1.15±0.63 | |
| **NF Mean±SD** | 0.75±0.81 | 0.92±0.56 | 1.53±0.99 | 1.49±0.80 | 0.79±0.63 | 1.01±0.74 | |
| **Tr Mean±SD** | 0.80±1.22 | 0.99±0.54 | 1.42±0.90 | 1.20±0.85 | 0.90±0.53 | 1.29±0.75\ | |
| **Tr_PF Mean±SD** | 0.68±0.64٭ | 1.01±0.50 | 1.43±0.91 | 1.21±0.80 | 0.82±0.55 | 1.15±0.71 | |
| **Tr_NF Mean±SD** | 0.84±0.81 | 0.97±0.62 | 1.52±1.05 | 1.44±0.85 | 0.91±0.59 | 1.11±0.82 | |
| **Tr_PF_NF Mean±SD** | 0.64±0.45 | 1.07±0.53 | 1.66±0.96٭ | 1.38±0.93 | 0.99±0.52 | 1.25±0.74 | |
| **PF_NF Mean±SD** | 0.67±0.52 | 1.04±0.49 | 1.78±0.93٭ | 1.43±0.90 | 0.98±0.49 | 1.23±0.76 | |

| **VASTUS MEDIALIS** | | | | | | |  |
| --- | --- | --- | --- | --- | --- | --- | --- |
|  | **DS1** | **SS1** | **SS2** | **DS2** | **SW1** | **SW2** | |
| **Average and SD Total AMP Scores for Amplitude Component** | | | | | | |  |
| **SS Mean±SD** | 0.47±0.28 | 0.91±0.69 | 0.99±0.90 | 0.57±0.28 | 0.60±0.21 | 1.09±0.91 | |
| **PF Mean±SD** | 0.83±0.55٭ | 1.29±0.79٭ | 1.92±1.43٭ | 0.53±0.27 | 0.62±0.23 | 1.11±1.00 | |
| **NF Mean±SD** | 0.70±0.52 | 1.23±0.66 | 2.17±1.63٭ | 0.52±0.27 | 0.60±0.28 | 1.08±.098 | |
| **Tr Mean±SD** | 0.68±0.52 | 1.10±0.65 | 1.82±1.40٭ | 0.47±0.30 | 0.62±0.36 | 1.11±1.11 | |
| **Tr_PF Mean±SD** | 0.70±0.54 | 1.10±0.67 | 2.04±1.39٭ | 0.51±0.26 | 0.60±0.33 | 1.10±0.96 | |
| **Tr_NF Mean±SD** | 0.68±0.48 | 1.10±0.70 | 2.24±1.53٭ | 0.51±0.26 | 0.61±0.26 | 1.17±0.92 | |
| **Tr_PF_NF Mean±SD** | 0.89±0.54٭ | 1.25±0.66٭ | 2.39±1.51٭ | 0.51±0.25 | 0.55±0.29 | 1.11±0.93 | |
| **PF_NF Mean±SD** | 0.96±0.57٭ | 1.23±0.64 | 2.34±1.35٭ | 0.50±0.28 | 0.62±0.18 | 1.13±1.01 | |
| **Average and SD Total AMP Scores for Timing Component** | | | | | | |  |
| **SS Mean±SD** | 0.92±0.39 | 1.42±0.50 | 1.50±1.08 | 0.64±0.39 | 0.63±0.22 | 0.95±0.72 | |
| **PF Mean±SD** | 0.85±0.33 | 1.59±0.82 | 1.92±1.36 | 0.75±0.47 | 0.68±0.44 | 1.15±0.73 | |
| **NF Mean±SD** | 0.78±0.41 | 1.57±0.73 | 2.04±1.51 | 0.71±0.47 | 0.66±0.29 | 0.98±0.78 | |
| **Tr Mean±SD** | 0.80±0.51 | 1.58±0.73 | 1.86±1.42 | 0.72±0.45 | 0.62±0.55 | 1.12±0.75 | |
| **Tr_PF Mean±SD** | 0.83±0.50 | 1.54±0.78 | 2.04±1.30٭ | 0.68±0.44 | 0.73±0.54 | 1.31±0.69 | |
| **Tr_NF Mean±SD** | 0.78±0.38 | 1.44±0.74 | 2.16±1.43 | 0.81±0.53 | 0.70±0.36 | 1.10±0.68 | |
| **Tr_PF_NF Mean±SD** | 0.79±0.33 | 1.51±0.79 | 2.32±1.25٭ | 0.72±0.47 | 0.58±0.43 | 1.26±0.70 | |
| **PF_NF Mean±SD** | 0.83±0.40٭ | 1.54±0.81 | 2.28±1.35٭ | 0.81±0.43 | 0.71±0.47 | 1.29±0.82 | |

| **GLUTEUS MEDIUS** | | | | | | |  |
| --- | --- | --- | --- | --- | --- | --- | --- |
|  | **DS1** | **SS1** | **SS2** | **DS2** | **SW1** | **SW2** | |
| **Average and SD Total AMP Scores for Amplitude Component** | | | | | | |  |
| **SS Mean±SD** | 0.98±0.77 | 2.04±1.34 | 1.32±0.63 | 1.82±1.70 | 1.01±0.85 | 1.54±1.39 | |
| **PF Mean±SD** | 0.93±0.67 | 0.90±0.61٭ | 0.61±0.49٭ | 0.95±0.76 | 0.79±0.53 | 1.68±1.39 | |
| **NF Mean±SD** | 0.71±0.60 | 1.22±0.77 | 0.67±0.65٭ | 1.15±0.95 | 0.64±0.65 | 1.27±1.03 | |
| **Tr Mean±SD** | 0.82±0.53 | 0.86±0.68٭ | 0.66±0.42٭ | 1.13±0.98 | 0.69±0.57 | 1.66±1.25 | |
| **Tr_PF Mean±SD** | 0.83±0.53 | 0.86±0.60٭ | 0.53±0.37٭ | 0.91±0.74 | 0.63±0.54 | 2.25±1.39 | |
| **Tr_NF Mean±SD** | 0.91±0.55 | 1.42±0.80 | 0.71±0.50٭ | 1.47±1.10 | 0.95±0.55 | 1.62±0.95 | |
| **Tr_PF_NF Mean±SD** | 0.85±0.63 | 1.10±0.88٭ | 0.77±0.57٭ | 1.03±0.80 | 0.82±0.67 | 2.72±1.48٭ | |
| **PF_NF Mean±SD** | 0.94±0.69 | 1.16±0.66 | 0.67±0.56٭ | 0.91±0.80 | 0.60±0.48 | 2.00±1.44 | |
| **Average and SD Total AMP Scores for Timing Component** | | | | | | |  |
| **SS Mean±SD** | 1.01±1.04 | 2.35±3.75 | 1.14±0.78 | 1.43±1.06 | 0.85±0.64 | 1.34±1.09 | |
| **PF Mean±SD** | 1.15±0.97 | 0.89±0.92 | 0.66±0.60٭ | 1.09±0.81 | 0.74±0.53 | 1.19±0.96 | |
| **NF Mean±SD** | 0.97±1.00 | 1.62±1.25 | 0.69±0.4 | 1.26±0.91 | 0.76±0.58 | 1.03±0.92 | |
| **Tr Mean±SD** | 1.12±0.69 | 0.93±1.00 | 0.55±0.32٭ | 1.19±0.75 | 0.79±0.62 | 1.30±0.98 | |
| **Tr_PF Mean±SD** | 0.80±0.62 | 0.86±0.77 | 0.52±0.33٭ | 0.94±0.63 | 0.72±0.55 | 1.68±1.09 | |
| **Tr_NF Mean±SD** | 0.81±0.69 | 1.97±1.52 | 0.74±0.50 | 1.55±0.98 | 1.07±0.62 | 1.36±0.89 | |
| **Tr_PF_NF Mean±SD** | 1.06±0.64 | 1.75±1.45 | 0.81±0.56 | 1.18±0.71 | 0.75±0.61 | 1.95±1.01٭ | |
| **PF_NF Mean±SD** | 1.20±1.28 | 1.38±1.17 | 0.71±0.51 | 1.02±0.71 | 0.62±0.50 | 1.43±1.06 | |
