# Supplementary material for: Variation of body weight supported treadmill training parameters during a single session can modulate muscle activity patterns in post-stroke gait
Source: Exp Brain Res. 2023 Jan 13;241(2):615–27. doi: 10.1007/s00221-023-06551-7 (PMC9895011; doi:10.1007/s00221-023-06551-7)
Supplement: Supplementary file 3 — Supplementary file3 (DOCX 46 kb) [file 221_2023_6551_MOESM3_ESM.docx]

Table 3. P-values FOR THERAPIST ASSISTED CONDITIONS COMPARED TO BASELINE

| **SOLEUS** | | | | | | |  |
| --- | --- | --- | --- | --- | --- | --- | --- |
|  | **DS1** | **SS1** | **SS2** | **DS2** | **SW1** | **SW2** | |
| **Amplitude Component** | | | | | | |  |
| **PF** | 0.254 | <0.001 | <0.001 | 0.15 | 0.369 | 0.054 | |
| **NF** | 0.611 | <0.001 | <0.001 | 0.339 | 0.14 | 0.001 | |
| **Tr** | 0.791 | 0.001 | 0.001 | 0.447 | 0.228 | 0.007 | |
| **Tr_PF** | 0.046 | <0.001 | <0.001 | 0.582 | 0.099 | 0.027 | |
| **Tr_NF** | 0.348 | 0.001 | <0.001 | 0.095 | 0.339 | 0.039 | |
| **Tr_PF_NF** | 0.002 | <0.001 | <0.001 | 0.614 | 0.149 | 0.021 | |
| **PF_NF** | 0.046 | 0.003 | <0.001 | 0.665 | 0.29 | 0.113 | |
| **Timing Component** | | | | | | |  |
| **PF** | 0.05 | 0.004 | 0.722 | 0.708 | 0.808 | 0.043 | |
| **NF** | 0.339 | 0.004 | 0.662 | 0.768 | 0.272 | <0.001 | |
| **Tr** | 0.106 | 0.038 | 0.222 | 0.197 | 0.174 | 0.001 | |
| **Tr_PF** | 0.251 | 0.023 | 0.593 | 0.863 | 0.05 | 0.108 | |
| **Tr_NF** | 0.235 | 0.018 | 0.95 | 0.131 | 0.48 | 0.017 | |
| **Tr_PF_NF** | 0.952 | 0.01 | 0.428 | 0.98 | 0.135 | 0.009 | |
| **PF_NF** | 0.374 | 0.064 | 0.428 | 0.737 | 0.313 | 0.22 | |

| **MEDIAL GASTROCNEMIUS** | | | | | | |  |
| --- | --- | --- | --- | --- | --- | --- | --- |
|  | **DS1** | **SS1** | **SS2** | **DS2** | **SW1** | **SW2** | |
| **Amplitude Component** | | | | | | |  |
| **PF** | 0.315 | 0.01 | 0.064 | 0.007 | 0.738 | 0.018 | |
| **NF** | 0.047 | 0.001 | 0.237 | 0.005 | 0.544 | 0.106 | |
| **Tr** | 0.009 | 0.004 | 0.024 | 0.003 | 0.681 | 0.638 | |
| **Tr_PF** | 0.851 | 0.011 | 0.041 | <0.001 | 0.304 | 0.043 | |
| **Tr_NF** | 0.033 | 0.001 | 0.082 | 0.002 | 0.299 | 0.417 | |
| **Tr_PF_NF** | 0.331 | 0.003 | 0.926 | <0.001 | 0.64 | 0.096 | |
| **PF_NF** | 0.568 | 0.019 | 0.23 | 0.019 | 0.732 | 0.278 | |
| **Timing Component** | | | | | | |  |
| **PF** | 0.005 | 0.09 | 0.23 | 0.482 | 0.524 | 0.053 | |
| **NF** | 0.026 | 0.237 | 0.199 | 0.262 | 0.959 | 0.629 | |
| **Tr** | 0.008 | 0.754 | 0.071 | 0.078 | 0.299 | 0.681 | |
| **Tr_PF** | 0.044 | 0.044 | 0.257 | 0.993 | 0.085 | 0.027 | |
| **Tr_NF** | 0.033 | 0.704 | 0.229 | 0.069 | 0.894 | 0.992 | |
| **Tr_PF_NF** | 0.562 | 0.15 | 0.562 | 0.34 | 0.145 | 0.038 | |
| **PF_NF** | 0.032 | 0.043 | 0.53 | 0.331 | 0.391 | 0.056 | |

| **MEDIAL HAMSTRINGS** | | | | | | |  |
| --- | --- | --- | --- | --- | --- | --- | --- |
|  | **DS1** | **SS1** | **SS2** | **DS2** | **SW1** | **SW2** | |
| **Amplitude Component** | | | | | | |  |
| **PF** | 0.004 | 0.281 | 0.299 | 0.531 | 0.009 | 0.505 | |
| **NF** | 0.29 | 0.481 | 0.224 | 0.264 | 0.57 | 0.158 | |
| **Tr** | 0.197 | 0.852 | 0.082 | 0.667 | 0.955 | 0.477 | |
| **Tr_PF** | 0.004 | 0.791 | 0.263 | 0.726 | 0.016 | 0.499 | |
| **Tr_NF** | 0.122 | 0.666 | 0.075 | 0.203 | 0.493 | 0.256 | |
| **Tr_PF_NF** | 0.001 | 0.331 | 0.4 | 0.562 | 0.079 | 0.911 | |
| **PF_NF** | 0.004 | 0.675 | 0.551 | 0.79 | 0.066 | 0.603 | |
| **Timing Component** | | | | | | |  |
| **PF** | 0.052 | 0.06 | 0.014 | 0.531 | 0.096 | <0.001 | |
| **NF** | 0.422 | 0.272 | 0.046 | 0.264 | 0.799 | 0.505 | |
| **Tr** | 0.627 | 0.073 | 0.014 | 0.667 | 0.562 | 0.27 | |
| **Tr_PF** | 0.925 | 0.032 | 0.008 | 0.726 | 0.046 | <0.001 | |
| **Tr_NF** | 0.055 | 0.203 | 0.006 | 0.203 | 0.104 | 0.142 | |
| **Tr_PF_NF** | 0.254 | 0.01 | 0.008 | 0.562 | 0.096 | <0.001 | |
| **PF_NF** | 0.2 | 0.046 | 0.062 | 0.79 | 0.112 | <0.001 | |

| **LATERAL HAMSTRINGS** | | | | | | |  |
| --- | --- | --- | --- | --- | --- | --- | --- |
|  | **DS1** | **SS1** | **SS2** | **DS2** | **SW1** | **SW2** | |
| **Amplitude Component** | | | | | | |  |
| **PF** | 0.001 | 0.658 | 0.754 | 0.294 | <0.001 | 0.854 | |
| **NF** | 0.049 | 0.02 | 0.405 | 0.704 | 0.013 | 0.206 | |
| **Tr** | 0.196 | 0.86 | 0.153 | 0.61 | 0.011 | 0.189 | |
| **Tr_PF** | 0.019 | 0.304 | 0.048 | 0.235 | 0.001 | 0.469 | |
| **Tr_NF** | 0.028 | 0.318 | 0.199 | 0.491 | 0.006 | 0.299 | |
| **Tr_PF_NF** | <0.001 | 0.256 | 0.232 | 0.357 | <0.001 | 0.224 | |
| **PF_NF** | 0.003 | 0.13 | 0.841 | 0.137 | 0.001 | 0.775 | |
| **Timing Component** | | | | | | |  |
| **PF** | 0.013 | 0.658 | 0.122 | 0.443 | 0.003 | 0.09 | |
| **NF** | 0.012 | 0.861 | 0.262 | 0.165 | 0.171 | 0.673 | |
| **Tr** | 0.005 | 0.681 | 0.034 | 0.829 | 0.505 | 0.86 | |
| **Tr_PF** | 0.043 | 0.235 | 0.005 | 0.809 | 0.145 | 0.011 | |
| **Tr_NF** | 0.004 | 0.262 | 0.136 | 0.91 | 0.478 | 0.349 | |
| **Tr_PF_NF** | 0.036 | 0.29 | 0.011 | 0.845 | 0.033 | 0.003 | |
| **PF_NF** | 0.007 | 0.587 | 0.17 | 0.689 | 0.046 | 0.009 | |

| **TIBIALIS ANTERIOR** | | | | | | |  |
| --- | --- | --- | --- | --- | --- | --- | --- |
|  | **DS1** | **SS1** | **SS2** | **DS2** | **SW1** | **SW2** | |
| **Amplitude Component** | | | | | | |  |
| **PF** | 0.938 | 0.164 | 0.86 | 0.21 | 0.357 | 0.505 | |
| **NF** | 0.026 | 0.808 | 0.852 | 0.006 | 0.627 | 0.466 | |
| **Tr** | 0.073 | 0.611 | 0.936 | 0.005 | 0.376 | 0.313 | |
| **Tr_PF** | 0.088 | 0.368 | 0.635 | 0.145 | 0.896 | 0.359 | |
| **Tr_NF** | 0.13 | 0.601 | 0.955 | 0.018 | 0.822 | 0.525 | |
| **Tr_PF_NF** | 0.296 | 0.006 | 0.406 | 0.458 | 0.796 | 0.586 | |
| **PF_NF** | 0.151 | 0.316 | 0.439 | 0.209 | 0.228 | 0.675 | |
| **Timing Component** | | | | | | |  |
| **PF** | 0.875 | 0.088 | 0.557 | 0.281 | 0.063 | 0.481 | |
| **NF** | 0.184 | 0.322 | 0.866 | 0.411 | 0.286 | 0.896 | |
| **Tr** | 0.348 | 0.561 | 0.688 | 0.067 | 0.95 | 0.851 | |
| **Tr_PF** | 0.857 | 0.105 | 1 | 0.159 | 0.067 | 0.555 | |
| **Tr_NF** | 0.204 | 0.304 | 0.896 | 0.411 | 0.601 | 0.525 | |
| **Tr_PF_NF** | 0.357 | 0.062 | 0.549 | 0.091 | 0.043 | 0.574 | |
| **PF_NF** | 0.367 | 0.501 | 0.638 | 0.367 | 0.036 | 0.99 | |

| **RECTUS FEMORIS** | | | | | | |  |
| --- | --- | --- | --- | --- | --- | --- | --- |
|  | **DS1** | **SS1** | **SS2** | **DS2** | **SW1** | **SW2** | |
| **Amplitude Component** | | | | | | |  |
| **PF** | 0.136 | 0.058 | 0.068 | 0.564 | 0.456 | 0.501 | |
| **NF** | 0.029 | 0.096 | 0.027 | 0.285 | 0.236 | 0.838 | |
| **Tr** | 0.177 | 0.053 | 0.05 | 0.581 | 0.256 | 0.871 | |
| **Tr_PF** | 0.203 | 0.009 | 0.065 | 0.681 | 0.531 | 0.505 | |
| **Tr_NF** | 0.076 | 0.285 | 0.08 | 0.982 | 0.246 | 0.569 | |
| **Tr_PF_NF** | 0.028 | 0.033 | 0.01 | 0.75 | 0.066 | 0.229 | |
| **PF_NF** | 0.031 | 0.095 | 0.011 | 0.39 | 0.338 | 0.592 | |
| **Timing Component** | | | | | | |  |
| **PF** | 0.02 | 0.29 | 0.031 | 0.361 | 0.061 | 0.387 | |
| **NF** | 0.106 | 0.767 | 0.01 | 0.122 | 0.699 | 0.82 | |
| **Tr** | 0.013 | 0.738 | 0.014 | 0.347 | 0.405 | 0.122 | |
| **Tr_PF** | 0.002 | 0.544 | 0.01 | 0.583 | 0.456 | 0.428 | |
| **Tr_NF** | 0.145 | 0.946 | 0.023 | 0.339 | 0.227 | 0.633 | |
| **Tr_PF_NF** | 0.079 | 0.405 | 0.002 | 0.975 | 0.066 | 0.271 | |
| **PF_NF** | 0.031 | 0.548 | 0.003 | 0.426 | 0.149 | 0.236 | |

| **VASTUS MEDIALIS** | | | | | | |  |
| --- | --- | --- | --- | --- | --- | --- | --- |
|  | **DS1** | **SS1** | **SS2** | **DS2** | **SW1** | **SW2** | |
| **Amplitude Component** | | | | | | |  |
| **PF** | 0.001 | 0.006 | <0.001 | 0.264 | 0.399 | 0.583 | |
| **NF** | 0.028 | 0.104 | 0.001 | 0.217 | 0.337 | 1 | |
| **Tr** | 0.059 | 0.357 | <0.001 | 0.043 | 0.993 | 0.768 | |
| **Tr_PF** | 0.043 | 0.317 | <0.001 | 0.158 | 0.7 | 0.939 | |
| **Tr_NF** | 0.044 | 0.256 | <0.001 | 0.29 | 0.327 | 0.652 | |
| **Tr_PF_NF** | <0.001 | 0.005 | <0.001 | 0.166 | 0.728 | 0.879 | |
| **PF_NF** | <0.001 | 0.122 | <0.001 | 0.412 | 0.51 | 0.619 | |
| **Timing Component** | | | | | | |  |
| **PF** | 0.256 | 0.027 | 0.04 | 0.544 | 0.142 | 0.327 | |
| **NF** | 0.009 | 0.044 | 0.046 | 0.638 | 0.096 | 0.814 | |
| **Tr** | 0.037 | 0.024 | 0.02 | 0.768 | 0.796 | 0.249 | |
| **Tr_PF** | 0.021 | 0.209 | 0.004 | 0.939 | 0.197 | 0.019 | |
| **Tr_NF** | 0.023 | 0.505 | 0.008 | 0.456 | 0.05 | 0.61 | |
| **Tr_PF_NF** | 0.009 | 0.264 | <0.001 | 0.574 | 0.675 | 0.055 | |
| **PF_NF** | 0.006 | 0.339 | 0.001 | 0.313 | 0.6 | 0.221 | |

| **GLUTEUS MEDIUS** | | | | | | |  |
| --- | --- | --- | --- | --- | --- | --- | --- |
|  | **DS1** | **SS1** | **SS2** | **DS2** | **SW1** | **SW2** | |
| **Amplitude Component** | | | | | | |  |
| **PF** | 0.992 | 0.001 | <0.001 | 0.009 | 0.53 | 0.199 | |
| **NF** | 0.206 | 0.02 | 0.001 | 0.086 | 0.102 | 0.6 | |
| **Tr** | 0.751 | <0.001 | <0.001 | 0.047 | 0.379 | 0.489 | |
| **Tr_PF** | 0.427 | <0.001 | <0.001 | 0.01 | 0.039 | 0.011 | |
| **Tr_NF** | 0.992 | 0.066 | <0.001 | 0.309 | 0.614 | 0.213 | |
| **Tr_PF_NF** | 0.513 | 0.003 | <0.001 | 0.016 | 0.224 | <0.001 | |
| **PF_NF** | 0.732 | 0.056 | 0.001 | 0.046 | 0.076 | 0.032 | |
| **Timing Component** | | | | | | |  |
| **PF** | 0.185 | 0.066 | 0.003 | 0.106 | 0.781 | 0.734 | |
| **NF** | 0.766 | 0.845 | 0.014 | 0.309 | 0.943 | 0.221 | |
| **Tr** | 0.025 | 0.029 | 0.001 | 0.145 | 0.911 | 0.94 | |
| **Tr_PF** | 0.908 | 0.015 | 0.001 | 0.026 | 0.586 | 0.05 | |
| **Tr_NF** | 0.877 | 0.558 | 0.014 | 0.943 | 0.072 | 0.614 | |
| **Tr_PF_NF** | 0.104 | 0.64 | 0.059 | 0.224 | 0.278 | 0.005 | |
| **PF_NF** | 0.278 | 0.29 | 0.034 | 0.23 | 0.317 | 0.265 | |
